# Analyses on Flavonoids and Transcriptome Reveals Key *MYB* Gene for Proanthocyanidins Regulation in *Onobrychis Viciifolia*

**DOI:** 10.3389/fpls.2022.941918

**Published:** 2022-06-24

**Authors:** Zhongzhiyue Jin, Wenbo Jiang, Yijing Luo, Haijun Huang, Dengxia Yi, Yongzhen Pang

**Affiliations:** Institute of Animal Sciences, Chinese Academy of Agricultural Sciences, Beijing, China

**Keywords:** *Onobrychis viciifolia*, flavonoids, proanthocyanidins, transcriptome, OvMYBPA2

## Abstract

*Onobrychis viciifolia* (sainfoin) is one of the most high-quality legume forages, which is rich in proanthocyanidins that is beneficial for the health and production of animals. In this study, proanthocyanidins and total flavonoids in leaves of 46 different sainfoin germplasm resources were evaluated, and it showed that soluble proanthocyanidin contents varied greatly in these sainfoin germplasm resources, but total flavonoids did not show significant difference. Transcriptome sequencing with high and low proanthocyanidins sainfoin resulted in the identification of totally 52,926 unigenes in sainfoin, and they were classed into different GOC categories. Among them, 1,608 unigenes were differentially expressed in high and low proanthocyanidins sainfoin samples, including 1,160 genes that were upregulated and 448 genes that were downregulated. Analysis on gene enrichment *via* KEGG annotation revealed that the differentially expressed genes were mainly enriched in the phenylpropanoid biosynthetic pathway and the secondary metabolism pathway. We also analyzed the expression levels of structural genes of the proanthocyanidin/flavonoid pathway in roots, stems, and leaves in the high proanthocyanidin sainfoin *via* RT-qPCR and found that these genes were differentially expressed in these tissues. Among them, the expression levels of *F3'5'H* and *ANR* were higher in leaves than in roots or stems, which is consistent with proanthocyanidins content in these tissues. Among *MYB* genes that were differentially expressed, the expression of *OvMYBPA2* was relatively high in high proanthocyanidin sainfoin. Over-expression level of *OvMYBPA2* in alfalfa hairy roots resulted in decreased anthocyanin content but increased proanthocyanidin content. Our study provided transcriptome information for further functional characterization of proanthocyanidin biosynthesis-related genes in sainfoin and candidate key *MYB* genes for bioengineering of proanthocyanidins in plants.

## Introduction

*Onobrychis viciifolia* L., also known as sainfoin, is a perennial legume of the *Onobrychis* genus, which is one of the main genus of the Fabaceae family (Carbonero et al., 2011). The centers of genetic diversity of sainfoin originate in southern Central Asia, particularly on the Anatolian plateau of Turkey, the districts of the Caucasus, the margins of the Caspian fringes, parts of Iran, and the mixed swards of Asia Minor (Bhattarai et al., 2016; Mora-Ortiz and Smith, 2018). Sainfoin has a long cultivation history and a wide distribution in temperate and subtropical regions of Europe, North America, Russia, Middle East, and north and northeast Africa (Carbonero et al., 2011; Amirahmadi et al., 2016). Sainfoin, which is abundant in high nutrition, is planted in 23 provinces/autonomous regions of China, such as Gansu, Xinjiang, and Inner Mongolia. Sainfoin has the characteristics of high nutritional value, good palatability, high yield, and strong nitrogen fixation ability, and it is an excellent animal feed resource. It was reported that the ruminant animals fed with sainfoin with moderate level of proanthocyanidins (also called condensed tannins) will reduce the occurrence of bloating and the emission of urinary nitrogen, increase absorption of amino acid (Min et al., 2003) and body weight (Waghorn, 2008), inhibit the damage of pathogens and parasites (Liu et al., 2013; Rivaroli et al., 2019), and promote the ability of nitrogen fixation in environment (Re et al., 2014).

Proanthocyanidins (in short PAs) is one large group of flavonoid compounds, and they were the most widely distributed secondary metabolites and have been widely found in many plant species (Xie and Dixon, 2005). Flavonoids play the important roles with various biological activities for plants, animals, and humans, including but not limited to antiviral, anti-pathogenic bacteria, anti-parasitic, anti-oxidative, anti-inflammatory, anti-allergenic, anti-tumor activities (Pietta, 2000; Kawai et al., 2007; Serafini et al., 2010; Mai et al., 2015; Farhadi et al., 2019; Kopustinskiene et al., 2020; Lalani and Poh, 2020). A variety of flavonoid components are present in plants, including flavonols, flavones, anthocyanins, and proanthocyanidins (Panche et al., 2016), and they are categorized based on the basic skeleton and different oxidation degree (Cheynier et al., 2013). HPLC-MS is an effective tool for the detection of targeted or non-targeted flavonoid compounds in plants (Wu et al., 2013). Based on HPLC-MS analysis, different flavonoid or phenolic compounds were reported in sainfoin. Phenolic compounds in young leaves, young petioles, stems, flower stalks, and flower buds of sainfoin were identified using LC-ESI-MS/MS (Regos et al., 2009). Another study isolated and identified phenolic acid and flavonoids in sainfoin using HPLC-DAD, including arbutin, gallic acid, catechin, epicatechin, epigallocatechin, and procyanidin B2 (Regos and Treutter, 2010). These studies revealed that sainfoin is rich in flavonoids, in particular proanthocyanidins in leaves of some sainfoin, which is rare in legume forages for the prevention of the lethal bloating disease.

Most studies on sainfoin have focused on the identification of phenolic components and the optimization of identification method, as well as evaluation of the bioactivities of related compounds, but the mechanisms regulating flavonoid biosynthetic pathway remain unknown in sainfoin. Biosynthesis pathway of flavonoids has been studied in many model or non-model plant species, including *Arabidopsis thaliana, Antirrhinum majus, Zea mays*, and *Ginkgo biloba* (Saito et al., 2013; Tohge et al., 2017; Fujino et al., 2018; Su et al., 2020), and these studies identified many biosynthetic genes (e.g., *CHS, CHI, F3H, F3'H, F3'5'H, DFR, ANS, ANR*, and *LAR*), as well as different types of transcription factor genes (Liu et al., 2015). Among them, the MBW complex comprising of MYB, bHLH, and WD40 proteins is the major regulatory complex for the regulation of flavonoid and proanthocyanidins pathway (Hichri et al., 2011; Chezem and Clay, 2016). Among the MBW complex, MYBs act as the leading players in the biosynthesis of flavonoids especially proanthocyanidins in seeds or in fruits in many plant species, such as *A. thaliana, Vitis vinifera, Lotus japonicus*, and *Medicago truncatula* (Baudry et al., 2004; Bogs et al., 2007; Yoshida et al., 2010; Verdier et al., 2012). Therefore, mining and identifying key *MYB* genes that regulate proanthocyanidin biosynthesis in leaves of sainfoin could be beneficial to the bioengineering of proanthocyanidins in other legume forages that lack proanthocyanidins in leave, such as the most important legume forage alfalfa (*Medicago sativa*).

In this study, we analyzed flavonoid and proanthocyanidins content from 46 sainfoin germplasm resources and found proanthocyanidin content varied greatly among them. These special sainfoin germplasm resources with high proanthocyanidins content could be used for breeding. In addition, we selected two of them with relatively high or low proanthocyanidin content for transcriptome analysis and identified a large number of candidate genes potentially involved in proanthocyanidins pathway. Among them, we characterized one MYB transcription gene *OvMYBPA2* and found its over-expression could increase proanthocyanidin content in *M. sativa* hairy roots, by regulating flavonoid flux. Our studies on the function of *OvMYBPA2* provide new clues for the regulation mechanism of proanthocyanidins in sainfoin, as well as new candidate gene for genetic breeding of forages with improved quality.

## Materials and Methods

### Plant Materials

The seeds of 46 sainfoin (*Onobrychis viciifolia*) germplasms (Supplementary Table 1) were stored at the Forage Germplasm Bank at the Institute of Animal Science, the Chinese Academy of Agricultural Sciences (IAS-CAAS), Beijing, China. The sainfoin plants were grown from seeds in the CAAS Experimental Station at Lang Fang, Hebei province, China in 2019. The mature leaves were collected during May of 2020 and dried for determination of the contents of proanthocyanidins and total flavonoids.

The seedling of 3-month-old sainfoin plant nos. 25 and 33 was grown in growth chamber (16-h/8-h light/dark, 24°C), and young leaves were collected for transcriptome sequencing and flavonoid profiling. The roots, stems, and leaves of sainfoin nos. 25 and 33 were collected and immediately frozen in liquid nitrogen and then kept at −80°C for RNA extraction and qPCR analyses.

The seeds of alfalfa (*Medicago sativa* L., cultivar Zhongmu no.1) used for the generation of transgenic hairy roots in this study were stored at IAS-CAAS.

### Extraction, Quantification, and Identification of Flavonoid Compounds

The extraction and determination of total flavonoids, proanthocyanidins, and anthocyanins were referred to our previous study with slight modifications (Pang et al., 2007, 2009). All reagents were purchased from Sinopharm Chemical Reagent Company (Beijing, China). About 100 mg of dried sainfoin leaves was extracted with 5 ml of 70% acetone (containing 0.5% acetic acid). After ultrasonication at 30°C for 30 min and centrifugation at 2,500 rpm for 10 min, the supernatant was obtained. The supernatant was then transferred into a 15-ml tube, and the residues were re-extracted two times as above. For the determination of soluble proanthocyanidins, 2 ml of supernatant was extracted with 2 ml chloroform to separate hydrophilic from hydrophobic compounds. After centrifugation at 2,500 rpm for 10 min, the upper layer was retained and extracted two times with an equal volume of chloroform. Then, the supernatant was extracted with 500 μl of n-hexane three times. The resulting lower phase was freeze-dried and re-dissolved in 70% acetone extraction solution, and this solution was stored at −20°C for subsequent determination of soluble PAs. The freeze-dried residues were used as a preliminary sample to determine the content of insoluble PAs part.

For the analyses of soluble PAs, 50 μl sample was added into 750 μl DMACA solution (0.2% [w/v] DMACA in methanol-3N HCl [1:1]), and the mixture was determined spectrophotometrically at 640 nm within 15 min. The content of soluble PAs was calculated according to the standard curve with catechin as standard.

For the analyses of insoluble PAs, the insoluble part was dissolved in 5 ml n-butanol-HCl (v/v:95/5). After ultrasonication at room temperature for 30 min and centrifugation at 2,500 rpm for 10 min, the supernatant was detected at 550 nm as A550-1. The supernatant was poured back into the precipitation solution and boiled for 1 h. The absorbance of supernatant was measured again at 550 nm after cool down to room temperature, which was recorded as A550-2. The absorbance difference obtained by subtracting A550-1 from A550-2 was calculated as insoluble PAs, according to the standard curve of procyanidin B1 (Pang et al., 2007, 2009).

For analyses of total flavonoids, the mature leaves of sainfoin or transgenic alfalfa hairy roots were ground in liquid nitrogen and lyophilized at −52°C for 18 h. About 20 mg of samples was extracted with 1 ml of 80% methanol, sonicated for 30 min, and then kept at 4°C for 12 h, following sonication again as above and then centrifuged at 10,000 rpm for 20 min. Afterward, 400 μl water, 30 μl of 5% NaNO_2_, 30 μl of 10% AlCl_3_, 200 μl of 1M NaOH, and 240 μl water were added sequentially to 100 μl of the supernatant. The absorptions of mixture of total 1 ml were measured spectrophotometrically at 510 nm. Absorbance values were converted into total flavonoids using a standard curve with quercetin.

The fresh young leaves of sainfoin were grinded and freeze-dried in a vacuum dryer. About 1 ml of 80% MeOH was added into 50 mg leaf sample, sonicated at room temperature for 30 min, and then stored at 4°C overnight. After sonication as above and centrifugation at 13,000 g for 10 min, the supernatants filtered through a 0.22-μm filter were used for subsequent LC-MS analysis. A total of three biological replicates were set up for each sample. For UPLC-MS analyses, chromatographic separation was performed on an Agilent HPLC 6500 system with a hybrid quadrupole time-of-flight (QTOF) Mass Spectrometer (Agilent Technologies, Santa Clara, CA, USA) at 280 nm using an Eclipse XDB-C18 reverse-phase column (4.6 mm ×150 mm, 5 μm). The mobile phases A (100% methanol) and stationary phase B (water containing 1% formic acid) were used at a flow rate of 0.5 ml/min. The parameters and experimental conditions of the instrument were as follows: re-equilibrium with 100% A for 10 min, A:B (v/v) gradient was 5:95 at 0 min, 80:20 at 16 min, 100:0 from 18 −20 min, and 5:95 at 21 min. The column temperature was set at 30°C and the injection volume was 15 μl555. The detection conditions of MS under negative ion (NI) mode were as follows: sample temperature: 350°C, scan range: 100–1,000 (m/z), desolvation gas (N_2_) flow: 800 L/h, cone gas flow: 50 L/h, cone voltage−60 V, and capillary voltage 2 kV for NI mode.

For analyses of anthocyanins, 50 mg of fresh hairy roots was extracted with 500 μl of 80% methanol (containing 0.1% hydrochloric acid), sonicated at room temperature for 30 min, centrifuged at 2,500 rpm for 10 min, and transferred the supernatant to a new tube. About 400 μl of supernatant was added into 2-ml centrifuge tube containing 400 μl water and 400 μl chloroform and the mixture was centrifuged as above. About 600 μl of supernatant was detected at 530 nm and the anthocyanin content was calculated as previously reported (Pang et al., 2007, 2009).

### RNA Extraction, cDNA Library Construction, High-Throughput Sequencing and Transcriptome *de novo* Assembly and Annotation

Young leaves of 3-month-old sainfoin plant nos. 25 and 33 were collected and used for RNA extraction by using PureLink RNA Mini Kit (Promega, Shanghai, China) according to the manufacturer's instructions. Nanodrop 2000 was used to detect the concentration and purity of RNA (A260/280:1.8-2.2; the concentration: ≥200 ng/μl). About 1% of agarose gel was used to detect RNA integrity, and the RNA integrity value (RIN > 8.0) of those samples was assessed using the RNA Nano 6000 Assay Kit of the Bioanalyzer 2100 system (Agilent Technologies, CA, USA). A total amount of 2 μg high-quality RNA per sample was used as input material for the RNA-seq carried out by Zhongxing Bomai Biotechnology Company (Beijing, China).

In details, mRNA purified from total RNAs was transcribed into cDNA using a SuperScript Double-Stranded cDNA Synthesis Kit (Invitrogen, Shanghai, China) with reverse transcriptase and random primers. Then, cDNA was combined into the Illumina Novoseq 6000 platform (Illumina, Shanghai, China). Since sainfoin has no reference genome as a control, all clean data were assembled *de novo* through Trinity software (http://trinityrnaseq.sourceforge.net/;version, number: trinityrnaseq-r2013-02-25). The longest sequence of clean reads from contigs was selected as the representative sequence of each gene for the follow-up transcriptome analyses. All the unigene sequences were aligned with the NR, Swissprot, KEGG, and COG databases using BLASTX to obtain the corresponding annotation information. Unigene sequences were deposited in the Sequence Read Archive (SRA) database at NCBI under Bioproject ID: PRJNA810561.

### Identification of Differentially Expressed Genes, Function Annotation, and Analysis of DEGs

Gene expression levels were calculated as fragments per kilobase of exon model per million mapped reads (FPKM) using Bowtie and RSEM (http://deweylab.biostat.wisc.edu/rsem/) (Li and Dewey, 2011). DEGs of these two samples were identified from standardized reads using programDeSeq2. The false discovery rate (FDR)-corrected *p*-value (≤0.05) was used as threshold for GO analysis and identification. FDR and fold change (FC) were also used for evaluation of DEGs. Padj < 0.05 and |log2(FC)| >1.5 were set as the cutoff value to identify DEGs. The heat map was plotted by TB tools (Chen et al., 2020a).

Gene function was annotated based on the following databases: NCBI non-redundant protein sequences (NR), Gene Ontology (GO), eukaryotic Clusters of Orthologous Groups of proteins (KOG), Clusters of Orthologous Groups of proteins (COG), Swiss-Prot (A manually annotated and reviewed protein sequence database), and KEGG Ortholog database. DEGs in KEGG pathways were conducted using FunRich software (version:3.1.3). Clean reads were used to analyze the expression level of the transcripts.

### Cloning and Vector Construction

The open reading frame of *OvMYBPA2* was amplified with *KOD* with high fidelity DNA polymerase, and cDNAs obtained from leaves of sainfoin no. 25. The primers OvMYBPA2F and OvMYBPA2R used for cloning of *OvMYBPA2* gene are listed in Supplementary Table 2. PCR condition was as follows: 94°C for 3 min, 35 cycles of 94°C for 30 s, 55°C for 30 s, 68°C for 1 min, and followed by a final extension of 68°C for 7 min. PCR products were, respectively, cloned into the Gateway Entry vector pENTR/D-TOPO and verified by sequencing. The resulting entry vector of pENTR/D-OvMYBPA2 was then transferred to the gateway plant transformation destination vector pK7WG2D.1 using the gateway LR reaction to generate pK7WG2D.1-OvMYBPA2 according to the manufacturer's instructions (Invitrogen, Carlsbad, USA).

### Analyses of *MYB* Gene Family and Phylogenetic Relationship

The deduced OvMYBPA2 (GenBank accession: OM929200) protein sequences, together with MYB proteins related to flavonoid synthesis from other legume plants (Supplementary Table 3), were used for multiple alignment with DNAMAN 9.0 software. For analyses of phylogenetic relationship, thirty-seven MYB proteins related to flavonoid synthesis from various plant species were aligned with DNAMAN software, and then, the phylogenetic tree was constructed by MEGA 11.0 software using the neighbor-joining (NJ) model with 1,000 bootstrap replications.

### Analyses of Gene Expression by Real-Time Quantitative PCR

Samples from roots, stems, and leaves of sainfoin or alfalfa hairy roots were ground in liquid nitrogen, and total RNAs were extracted from different tissues using Eastep® Super total RNA Extraction Kit (Promega, Shanghai, China) according to the manufacturer's instructions. The first-stand cDNA was synthesized from 500 ng total RNAs by using FastKing gDNA Dispelling RT SuperMix (Tiangen, Beijing, China) according to the manufacturer's protocols. The RT-qPCRs were carried out on ABI 7500 real-time Detection System (Applied Biosystems, USA) using a 2× RealStar Green Fast Mixture (GeneStar, Being, China). Actin-related protein 4A genes were used as house-keeping gene as control in the RT-qPCR. The RT-qPCR conditions were as follows: 95°C for 2 min, 40 cycles of 95°C for 15s and 60°C for 34s, 95°C for 15s, 60°C for 1 min, 95°C for 15s, and 60°C for 15s. Each reaction was performed with three biological replicates, and the relative transcript levels were calculated compared to the internal control using the 2^−ΔΔ*CT*^ method. The data were presented as means ± standard. The primer used in this study was designed using the Premier 5.0 software, and primer sequences are listed in Supplementary Table 2.

### Generation and Identification of Transgenic Alfalfa Hairy Roots Mediated by *Agrobacterium*

The pK7WG2D.1-OvMYBPA2 plasmids confirmed by sequencing were transformed into the *Agrobacterium rhizogenes* strain Arqual I. The seeds of alfalfa were treated in sequence with concentrated sulfuric acid, sodium hypochlorite, and sterile water, spread flat on MS medium, and vernalized in a refrigerator at 4°C for 3 days. The hypocotyl parts were infected with Arqual I containing the above-mentioned plasmids and incubated on the F agar medium until the generation of hairy roots. The hairy roots were then transferred onto B5 medium containing 50 mg/L kanamycin for selection. Hairy roots of about 90 days old were ground in liquid nitrogen, and DNA was extracted with modified CTAB method (Drabkova, 2014). PCRs for the identification of positive hairy root lines were performed with *Taq* DNA polymerase with the same above PCR condition for gene cloning. The transgenic hairy roots of 90 days old were confirmed by PCR and RT-qPCR, and the positive lines were selected for analysis of anthocyanins, soluble PAs, and total flavonoids using the above-mentioned methods.

## Results

### Analyses of Proanthocyanidins and Total Flavonoids in Leaves of Sainfoin

During the quality evaluation of sainfoin, main flavonoids including soluble PAs, insoluble Pas, and total flavonoids were determined spectrophotometrically in 46 germplasm resources collected from home and abroad (Supplementary Table 1). It was revealed that soluble PA contents in leaves of 46 sainfoin germplasm resources ranged from 0 to 55.041 mg/g (Table 1). Among them, soluble PA contents in five of them were more than 45 mg/g (nos. 7, 19, 23, 25, and 32) (Table 1), whereas no PAs were detected in another four of them (nos. 21, 33, 39, and 40) (Table 1). Measurement on insoluble PAs content showed that it ranged from 0.506 to 4.354 mg/g, and the insoluble PAs did not show significant difference as soluble PAs among 46 germplasm resources (Table 1). We also determined total flavonoids and found that it ranged from 0.091 to 0.396 mg/g (Table 1). These data showed that soluble PAs content varied significantly in leaves of these 46 sainfoin germplasm resources, whereas insoluble PAs content and total flavonoid content did not (Table 1).

**Table 1:** Content of soluble PAs, insoluble PAs, and total flavonoid determined in leaves of 46 sainfoin germplasm resources.

**Sample ID**	**Soluble PAs (mg/g)**	**Insoluble PAs (mg/g)**	**Total flavonoid (mg/g)**
1	0.231 ± 0.189	0.520 ± 0.059	0.225 ± 0.009
2	27.319 ± 0.418	0.731 ± 0.082	0.180 ± 0.004
3	24.334 ± 0.837	1.339 ± 0.097	0.184 ± 0.009
4	17.461 ± 1.140	1.005 ± 0.056	0.129 ± 0.001
5	8.626 ± 1.621	0.653 ± 0.051	0.232 ± 0.006
6	18.785 ± 1.332	0.506 ± 0.043	0.127 ± 0.005
7	54.752 ± 1.846	3.412 ± 0.374	0.326 ± 0.010
8	14.343 ± 0.532	0.506 ± 0.043	0.132 ± 0.001
9	30.786 ± 1.766	0.731 ± 0.082	0.207 ± 0.010
10	13.477 ± 0.318	3.975 ± 0.327	0.162 ± 0.004
11	25.646 ± 3.515	0.959 ± 0.046	0.299 ± 0.005
12	39.766 ± 1.611	1.121 ± 0.045	0.267 ± 0.012
13	10.323 ± 1.425	2.157 ± 0.141	0.205 ± 0.010
14	33.675 ± 1.367	1.186 ± 0.030	0.201 ± 0.012
15	12.080 ± 0.454	0.880 ± 0.117	0.176 ± 0.007
16	34.879 ± 1.584	2.790 ± 0.134	0.288 ± 0.013
17	42.691 ± 3.202	1.110 ± 0.074	0.182 ± 0.012
18	22.902 ± 0.683	1.113 ± 0.076	0.213 ± 0.007
19	52.970 ± 0.445	2.249 ± 0.092	0.248 ± 0.009
20	12.730 ± 0.479	2.367 ± 0.146	0.161 ± 0.004
21	0.000	0.618 ± 0.050	0.091 ± 0.005
22	23.046 ± 1.445	0.885 ± 0.022	0.306 ± 0.002
23	48.252 ± 1.643	1.339 ± 0.097	0.392 ± 0.005
24	27.416 ± 1.372	2.249 ± 0.092	0.227 ± 0.005
**25**	**49.491** **±2.647**	**0.767** **±0.052**	**0.277** **±0.006**
26	15.427 ± 1.096	1.121 ± 0.045	0.208 ± 0.001
27	22.649 ± 1.541	1.113 ± 0.076	0.133 ± 0.002
28	36.179 ± 3.080	1.571 ± 0.142	0.237 ± 0.010
29	33.061 ± 2.455	1.005 ± 0.056	0.193 ± 0.014
30	23.082 ± 0.900	1.467 ± 0.084	0.164 ± 0.006
31	33.964 ± 1.148	1.571 ± 0.142	0.233 ± 0.005
32	55.041 ± 2.497	1.262 ± 0.094	0.294 ± 0.005
**33**	**0.000**	**0.608** **±0.044**	**0.241** **±0.007**
34	3.630 ± 0.280	1.010 ± 0.039	0.199 ± 0.006
35	41.210 ± 3.531	3.082 ± 0.131	0.364 ± 0.013
36	42.450 ± 0.639	2.735 ± 0.045	0.255 ± 0.004
37	18.340 ± 1.018	1.110 ± 0.074	0.249 ± 0.006
38	6.279 ± 1.165	0.579 ± 0.051	0.190 ± 0.003
39	0.000	1.071 ± 0.189	0.121 ± 0.005
40	0.000	1.634 ± 0.103	0.205 ± 0.004
41	0.537 ± 0.388	0.618 ± 0.007	0.228 ± 0.006
42	40.765 ± 2.430	2.690 ± 0.156	0.228 ± 0.006
43	21.674 ± 1.575	4.354 ± 0.298	0.233 ± 0.003
44	6.965 ± 0.353	1.495 ± 0.065	0.173 ± 0.002
45	21.806 ± 0.461	1.262 ± 0.094	0.273 ± 0.011
46	8.590 ± 0.788	1.186 ± 0.030	0.196 ± 0.004

*Data are presented as mean ± SD, with biological triplicates. Nos. 25 and 33 that were bold respectively represent sainfoin samples with high or low proanthocyanidins content for subsequent analyses*.

A total of four sainfoin germplasm resources with relative higher soluble PAs content (nos. 7, 19, 25, and 32), and another four (nos. 21, 33, 39, and 40) with relatively lower soluble PAs content, were selected and their leaves were tested at different sampling times. Among them, the soluble PA content in nos. 25 and 33 maintained stable, and they thus were selected for further analyses as high PA sample (no. 25, HPAs) and low PA sample (no. 33, LPAs).

The composition of flavonoids in leaves of sainfoin nos. 25 and 33 was further subjected for LC-MS analyses. Several peaks were identified and their content in these two samples was different (Supplementary Table 4; Supplementary Figure 1). Combining UV spectrograms, mass spectra, and previous reports (Regos et al., 2009; Regos and Treutter, 2010), the contents of eight compounds, namely, quercetin-3-*O*-rutinoside, kaempferol-3-*O*-rutinoside, kaempferol, quercetin, L-tryptophan, *p*-coumaroylquinic acid, myricetin 3-*O*-rhamnoglucoside, and caffeoylquinic acid, were different in these two samples (Supplementary Table 4). Among them, four compounds, including quercetin-3-*O*-rutinoside, kaempferol-3-*O*-rutinoside, quercetin, and kaempferol, were higher in no. 25 than in no. 33, whereas quercetin, L-tryptophan, myricetin 3-*O*-rhamnoglucoside, and caffeoylquinic acid were only found in no. 25. A total of five remaining peaks could not be identified based on available data, and their contents in nos. 25 and 33 were different (Supplementary Table 4). Meanwhile, no monomers, dimers, or other low molecular oligomers were identified in leaves of these two sainfoin samples by LC-MS under present detection condition (Supplementary Figure 1).

### Transcriptome Sequencing and Annotation

In this study, Illumina Hi-Seq paired-end sequencing technology was used to analyze the transcriptome of sainfoin leaves. Total RNAs were extracted and assessed, and no. 25 with high PAs and no. 33 with low PAs were established and sequenced with triplicates. As a result, a total of more than 40 billion high-quality clean reads after filtration were produced. The Q20 and Q30 of each sample were not less than 98.3 and 94.5%, respectively, and the GC content of each samples was between 43 and 45% (Supplementary Table 5). The clean reads were assembled by Trinity software to 52,926 transcripts with an average length of 1,063 bp and an N50 contig size of 1,587 bp after removing redundant transcripts, with the GC content of assembled unigenes of 40.14% (Supplementary Table 5). The length of the largest transcript and smallest transcript was, respectively, 14,543 and 301 bp. The length of N50 was higher than the average length of assembled unigenes (Supplementary Table 6). These data indicated that the quality of this sequencing met the requirements for subsequent transcriptome analysis.

**Figure 1:**
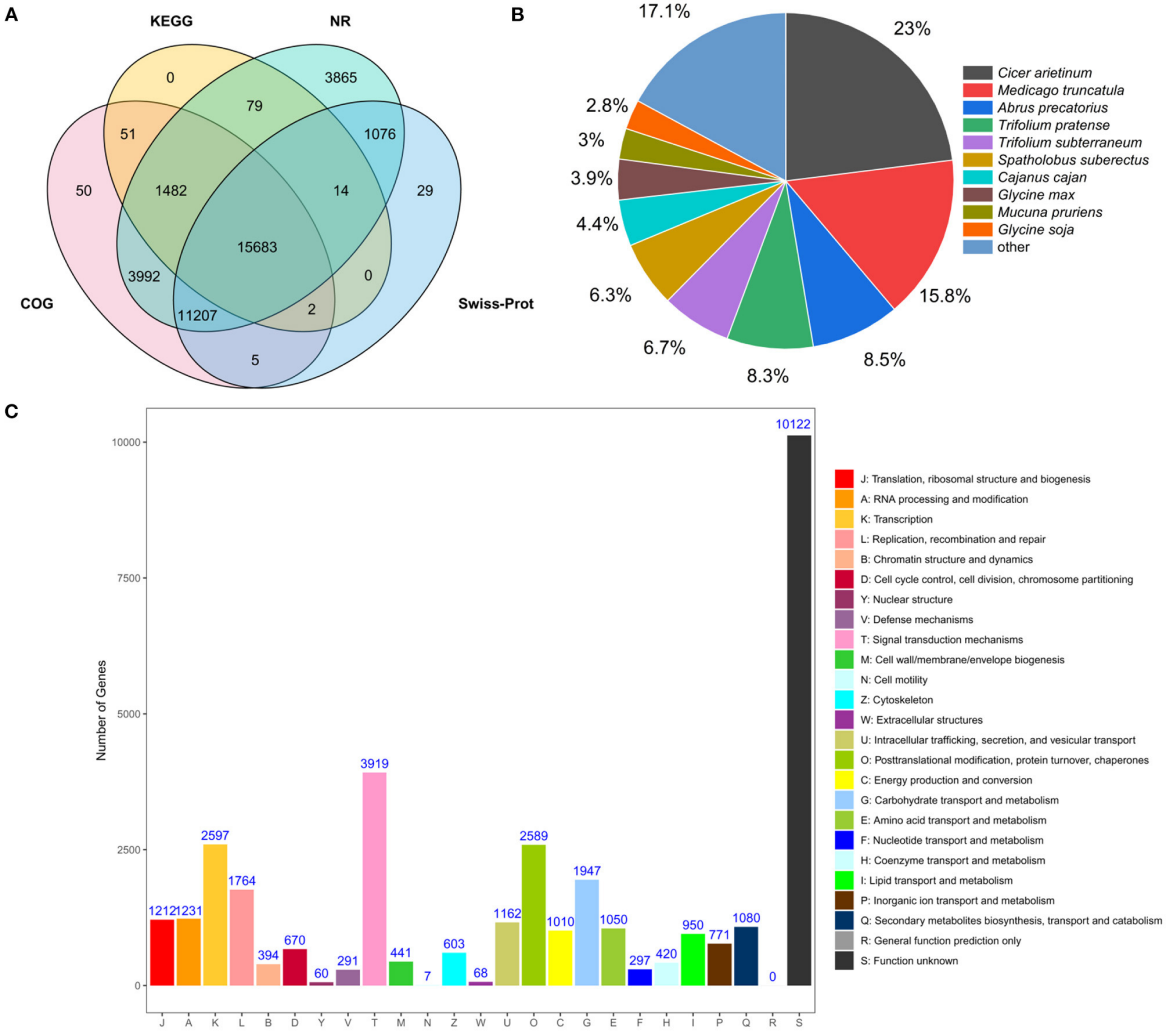
Analyses of transcriptome data from leaves of sainfoin. **(A)** Venn diagram showing the number of unigenes co-annotated to KEGG, NR, COG, and Swiss-Prot database. **(B)** NR annotation shows the distribution of homologous species for unigenes of *O. viciifolia*. Compared to the NR database, the area of the sector represents the proportion of unigenes that are homologous to the corresponding species. **(C)** COG classification of unigenes of *O. viciifolia*. The horizontal axis represents 25 functional categories, and every category was displayed with different color bars, which are denoted by capital letters A~Z. The vertical axis indicates the number of unigenes with corresponding functional categories.

All unigenes in accordance with the public databases NR, COG, KOG, KEGG, GO, Swisss-Prot were searched using BLASTX (E-value <10^−5^) for functional annotations. A total of 52,926 unigenes (100%) were annotated in the six databases. A total of 37,398 transcripts (70.6%) were significantly matched with NR database, and 32,472 (61.4%), 18,743 (35.4%), 17,312 (32.7%), 17,912 (33.8%), and 28,016 (52.9%) unigenes matched with COG, KOG, KEGG, GO, Swiss-Prot databases, respectively (Supplementary Table 7). It can be found that some unigenes could not be annotated with these database, and it is possible that these unannotated unigenes are unique to sainfoin and require more advanced sequencing techniques for further analyses. A Venn diagram showed that a total of 15,683 unigenes were co-annotated in KEGG, NR, COG, and Swiss-Prot databases (Figure 1A).

By comparing with the NR library, the annotated transcript of sainfoin was related to the following species with different transcriptome amount and percentage: *Cicer arietinum* (8,609, 23%), *Medicago truncatula* (5,913, 15.8%), *Abrus precatorius* (3,194,8.5%), *Trifolium pratense* (3,101, 8.3%), *Trifolium subterraneum* (2,515,6.7%), and *Spatholobus suberectus* (2,369, 6.3%) (Figure 1B). The results revealed that sainfoin had a relatively high homology with *C. arietinum* and *M. truncatula*, and they both belong to the legume family as sainfoin.

Using the GO database, clustering analysis of gene functions was annotated and classified according to the three aspects of biological process (BP), cellular component (CC), and molecular function (MF), and these three terms were mainly categorized into 60 subcategories. For the BP category, organic substance metabolic process (10,167), cellular metabolic process (10,142), primary metabolic process (9,514), and nitrogen compound metabolic process (8,558) were the most dominant subcategories (Supplementary Figure 2). In the subcategories of cellular components, genes were mainly annotated in organelle (11,620), cytoplasm (9,719), membrane (5,975), and cell periphery (4,136). The most enriched terms in molecular function were transferase activity (3,599), organic cyclic compound binding (3,570), heterocyclic compound binding (3,553), and hydrolase activity (3,526, Supplementary Figure 2).

For COG annotation, a total of 32,472 unigenes were divided to 25 groups. Signal transduction mechanism (T, 3,919) was the most abundant group (Figure 1C). According to the classification results, 1,080 genes were annotated to the group of secondary metabolites biosynthesis, transport, and catabolism (Q), and it can be hypothesized that these genes may be closely associated with metabolome variability in the leaves of sainfoin (Figure 1C).

Analysis of KEGG pathway genes was also performed, and a total of 17,312 unigenes (32.7%) were mapped to KEGG database. These unigenes were divided into five groups, namely, metabolism, genetic information processing, environmental information processing, cellular processes, and organismal systems, and they were further divided into 32 pathways (Supplementary Figure 3). Among these, carbohydrate metabolism (1,360), translation (1,147), signal transduction (2,682), transport and catabolism (1,110), and immune system (1,074) were the top five subcategories. Among these pathways, 124 and 17 genes were, respectively, associated with phenylpropanoid biosynthesis and flavonoid biosynthesis, and ko00940 was the most abundant pathway with enriched genes in phenylpropanoid biosynthesis (Supplementary Figure 3).

### Analyses of Differentially Expressed Genes Between HPAs and LPAs Sainfoin

To investigate DEGs that may be related to flavonoid metabolism between HPAs and LPAs, especially those related to PA biosynthesis, we performed a comparative analysis of the transcriptome of these two samples. The intergroup correlation values were not less than 0.93 among these two samples with triplicates (Supplementary Table 8), indicating that these six samples have high correlation and the subsequent analysis on differentially expressed genes was reliable.

**Figure 2:**
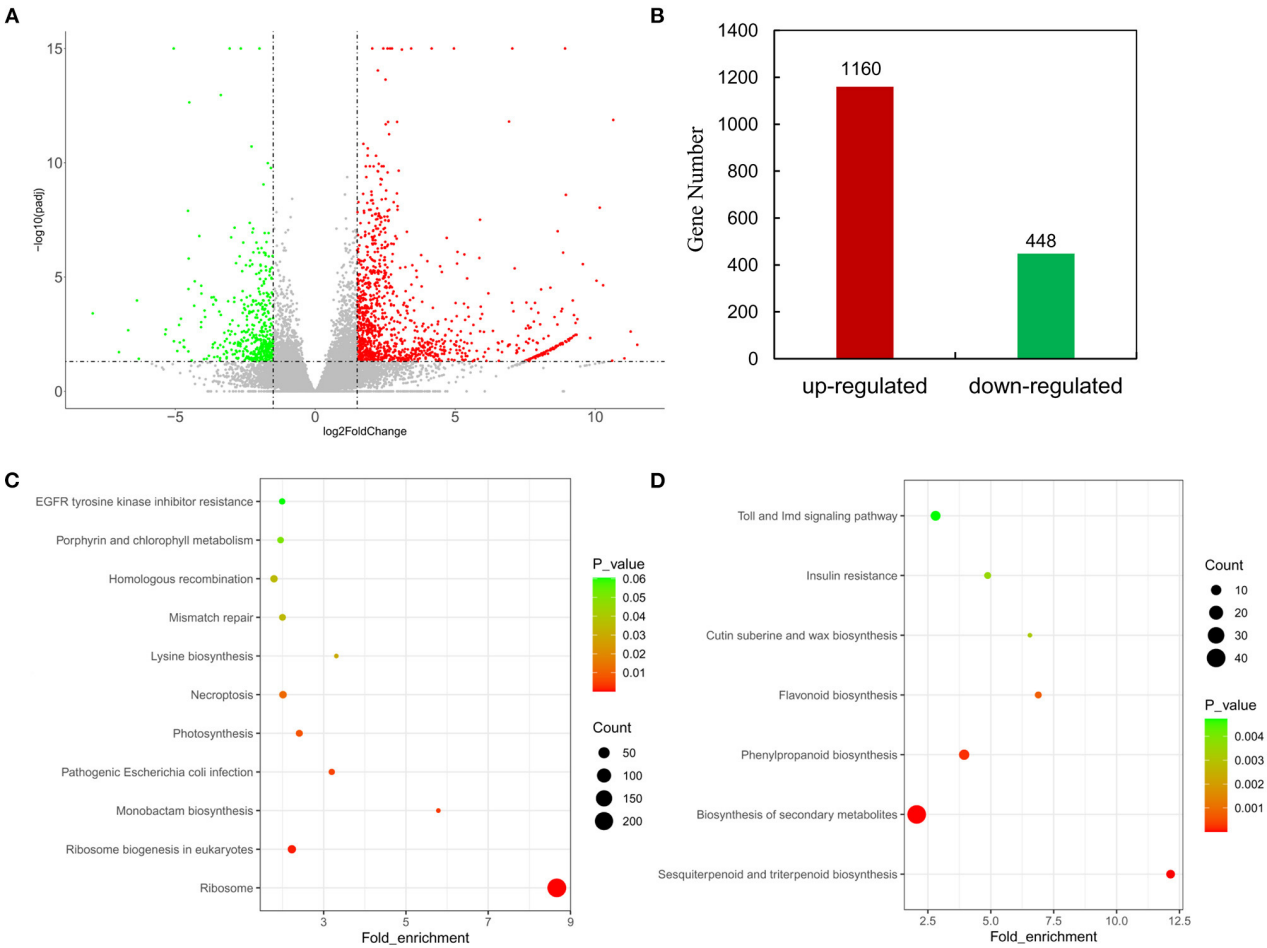
Analyses on DEGs in leaves of two *O. viciifolia* samples. **(A)** Volcano plots of DEGs. The horizontal axis represents the values of Log_2_FoldChange of DEGs in the samples of high PAs and low PAs groups, and the vertical axis represents the statistical significance of differences with *p*-value. Red represents upregulated DEGs and green represents downregulated DEGs. **(B)** Gene number of upregulated DEGs and downregulated DEGs. **(C)** Enrichment analyses of KEGG function of upregulated DEGs with 1,160 genes. **(D)** Enrichment analyses of KEGG function of downregulated DEGs with 448 genes. The horizontal axis is the fold enrichment factor, and the vertical axis is the KEGG pathway. The bubbles represent the number of genes. Different colors represent significance of differences (*p*-value).

The distribution of differences in gene expression levels was determined between HPAs and LPAs by constructing a volcano plot (Figure 2A). |Log2(fold change)| ≥1.5 and Padj ≤0.05 were set as thresholds to screen significant differentially expressed genes, and it was found that 1,608 genes were differentially expressed between the transcriptome of HPAs and LPAs, including 1,160 genes (expression level in HPAs/LPAs) that were upregulated and 448 genes that were downregulated (Figure 2B).

To analyze the major functional terms of these DEGs, we performed GO enrichment analysis with both upregulated and downregulated genes, respectively. Protein-containing complex, gene expression, ribonucleoprotein complex, structural molecule activity, and ribosome were the top five terms of GO enrichment analysis for the upregulated genes (Supplementary Figure 4). The top four terms of GO enrichment analysis for the downregulated genes were anion transmembrane transport, anion transmembrane transporter activity, organic anion transport, and anion transport (Supplementary Figure 5).

To analyze potential pathways involved in flavonoid metabolism, KEGG enrichment analysis was performed with all DEGs. KEGG enrichment analysis with the fold enrich factor, Q-value, and the count of genes in a given pathway were shown in bubble plots (Figures 2C,D). A total of 1,160 upregulated genes were mapped to 244 pathways and mainly enriched in ribosome (218), ribosome biogenesis in eukaryotes (18), pathogenic Escherichia coli infection (7), photosynthesis (10), and necroptosis (13, Figure 2C). All 448 downregulated genes were mapped to 132 pathways and mainly annotated to biosynthesis of secondary metabolites (40), sesquiterpenoid and triterpenoid biosynthesis (7), phenylpropanoid biosynthesis (10), and flavonoid biosynthesis (5, Figure 2D). The KEGG analysis for the downregulated genes showed that HPAs sainfoin had more DEGs enriched in secondary metabolite biosynthesis pathway and phenylpropanoid biosynthesis pathway than in flavonoid synthesis pathway compared with LPAs sainfoin. This result suggested that the PAs content in the leaves of HPAs sainfoin might be due to the downregulated of DEGs in the flavonoid synthesis pathway.

### Analysis of Differentially Expressed Genes Associated With PA Pathway

To understand the mechanism of flavonoid synthesis in sainfoin, we analyzed and categorized genes involved in flavonoid pathway from the transcriptome data and showed the expression levels of several representative structural genes in HPAs and LPAs sainfoin. Gene expression patterns of structural gene including *CHS, CHI, F3H, F3'H, F3'5'H, DFR, ANS, LAR*, and *ANR* were represented by the FPKM values and Log_2_FC (Supplementary Table 9). According to these data, it was obviously that the structural genes related to flavonoids biosynthesis had different expression patterns between HPAs and LPAs (Supplementary Table 9). It was found that upstream genes of the flavonoid pathway were mostly downregulated, such as *CHS* (TRINITY_DN1298_c0_g1_i1, TRINITY_DN613_c0_g1_i2, TRINITY_DN20751_c0_g1_i1, TRINITY_DN320_c0_g1_i4), *CHI* (TRINITY_DN12115_c0_g1_i1, TRINITY_DN586_c0_g2_i3), *F3H* (TRINITY_DN4402_c0_g1_i1), *F3'H* (TRINITY_DN1854_c0_g1_i3), and *F3'5'H* (TRINITY_DN2008_c0_g1_i3) (Supplementary Table 9). However, several downstream genes were upregulated genes such as *DFR* (TRINITY_DN282_c0_g1_i15), *LAR* (TRINITY_DN11234_c1_g1_i2), and *ANR* (TRINITY_DN293_c0_g2_i1) in the later flavonoid pathway for PA biosynthesis (Supplementary Table 9).

**Figure 3:**
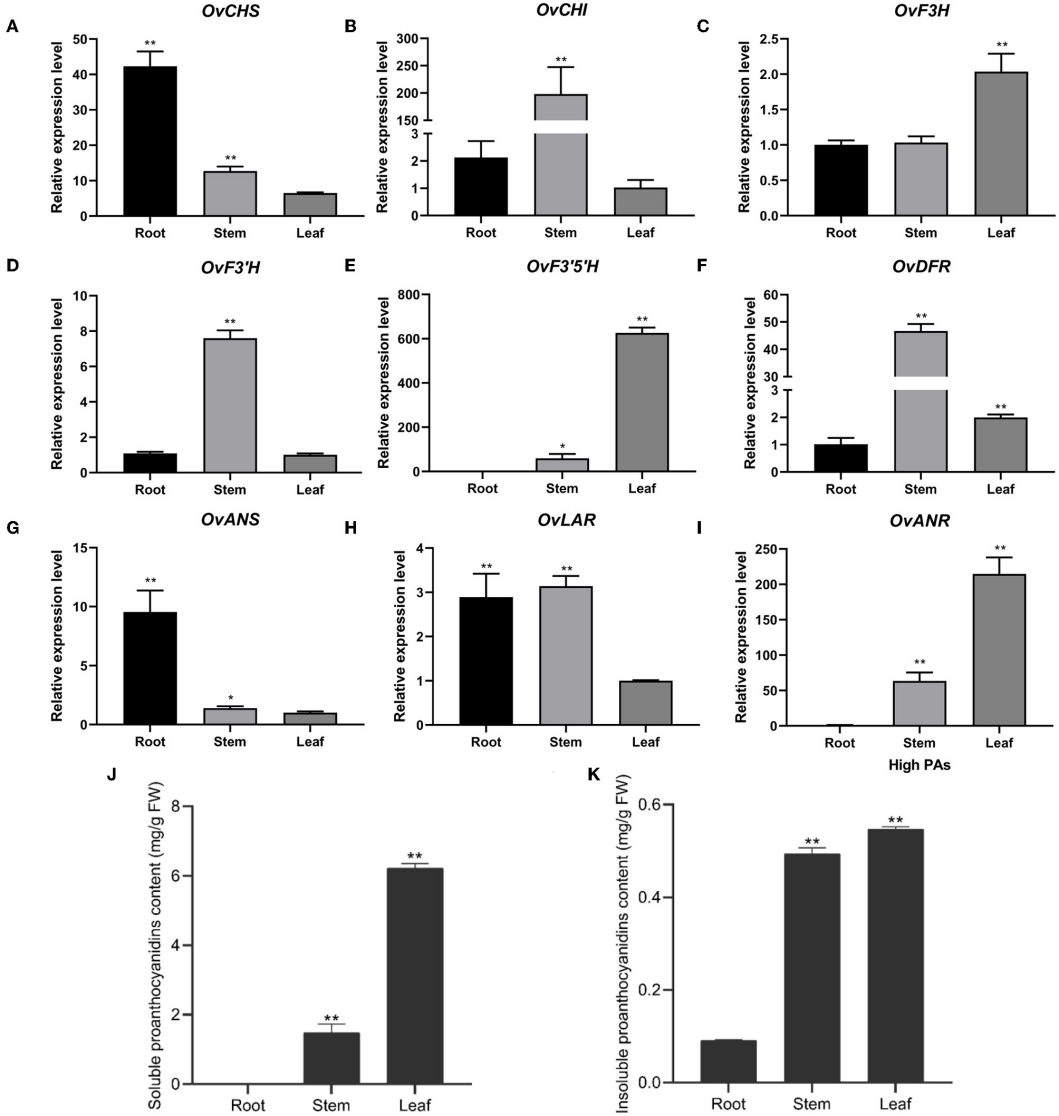
Relative expression levels of pathway genes in different tissues of *O. viciifolia*. The horizontal axis is different tissues (root, stem, and leaf) of HPAs sainfoin, and the vertical axis is relative expression levels. *OvCHS* **(A)**, *OvCHI* **(B)**, *OvF3H* **(C)**, *OvF3'H* **(D)**, *OvF3'5H* **(E)**, *OvDFR* **(F)**, *OvANS* **(G)**, *OvLAR* **(H)**, and *OvANR* **(I)**. **(J,K)** Soluble **(J)** and insoluble proanthocyanidins **(K)** content in root, stem, and leaf of HPAs sainfoin. Error bar depicts the standard error of mean ± SD of three biological replicates. Significance of differences was represented with one asterisk (*p* < 0.05) and two asterisks (*p* < 0.01).

To further investigate the expression of nine structural genes in different tissues, we analyzed their expression level using RT-qPCR in the roots, stems, and leaves of sainfoin with HPAs. It was shown that the expression of *CHS* and *ANS* shared a similar expression pattern which were both higher in roots than in stems and leaves (Figures 3A,G). *CHI, F3'H, DFR*, and *LAR* were highly expressed in stems than in roots and leaves (Figures 3B,D,F,H), and the expression levels of *F3H, F3'5'H*, and *ANR* were higher in leaves than in stems or roots (Figures 3C,E,I). It was worth noting that the expression levels of *F3'5'H* and *ANR* were relatively higher than other genes, in particular in leaves (Figures 3E,I). In addition, we also analyzed both soluble PAs and insoluble PAs in roots, stems, and leaves of in this sainfoin and found that both soluble and insoluble PA contents were higher in leaves than in roots or stems (Figures 3J,K). It was thus speculated that *F3'5'H* and *ANR* are the key genes for PAs biosynthesis in leaves of sainfoin.

**Figure 4:**
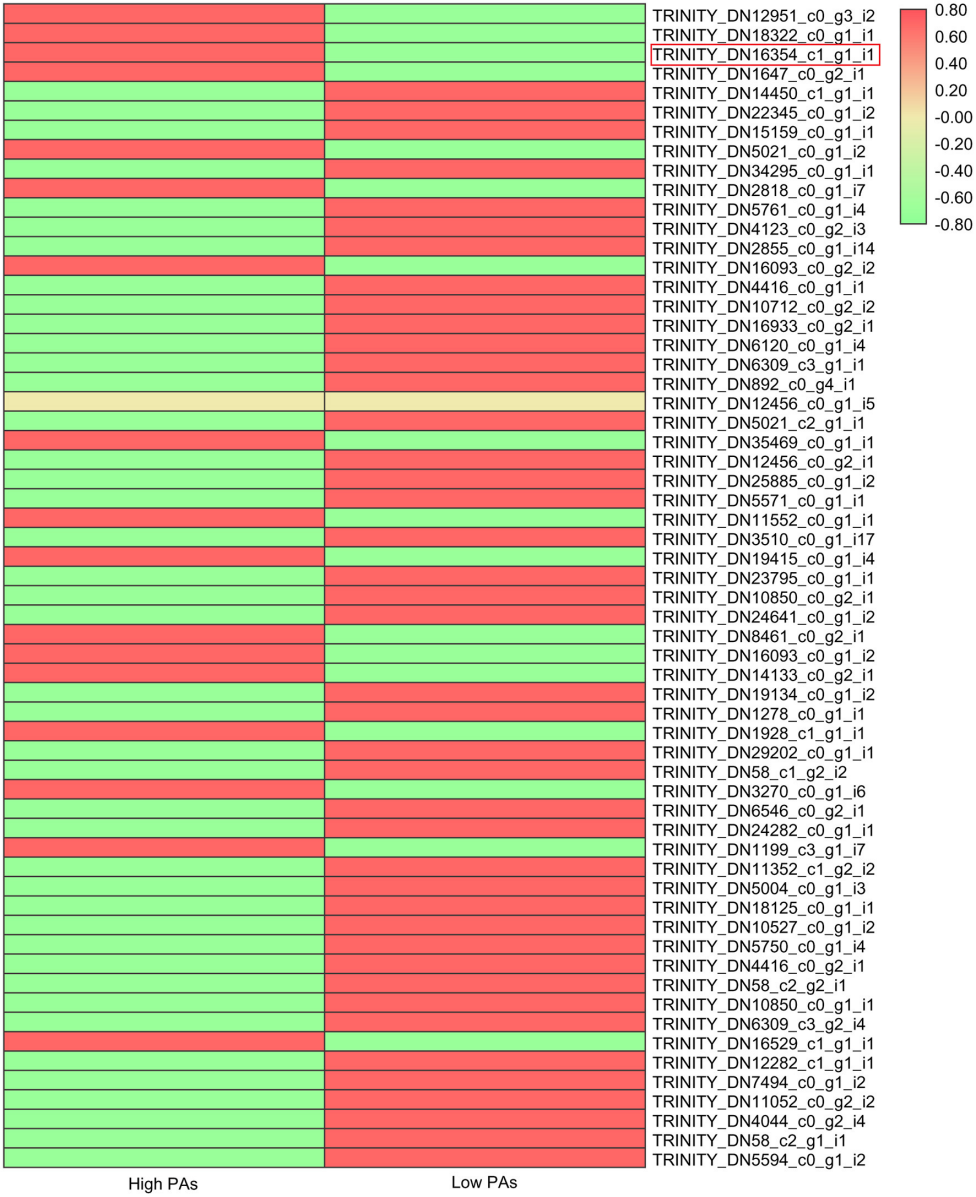
Expression levels of MYB transcription factor genes in two sainfoin samples. The heat map was drawn by TBtools with the Log_2_FPKM values of 60 MYBs in *O. viciifolia* with high PAs and low PAs. Different colors depict different expression levels, red means higher expression levels, and green means lower expression levels. Red box represents the MYB named as *OvMYBPA2* that was subsequently cloned and functionally validated in this study.

Many studies have shown that flavonoid pathway was mainly regulated by transcription factor complex composing of MYB, bHLH, and WD40. Hence, we also investigated the expression levels of all putative MYB, bHLH, and WD40 genes that were differentially expressed in two sainfoin samples (Figure 4; Supplementary Figures 6, 7). The expression levels of total 60 *MYBs*, 113 *bHLHs*, and 19 *WD40* genes were compared and shown in heat map (Figure 4; Supplementary Figures 6, 7). Among the MBW complex proteins, MYB is the key player by controlling the biosynthesis of PAs *via* enhancing or inhibiting the expression of structural genes (Hichri et al., 2011); therefore, we focused on *MYB* genes and found that 17 *MYB* genes were upregulated in HPAs when compared to LPAs (Figure 4), suggesting that these TFs may be associated with PAs biosynthesis in sainfoin. Among them, one unigene (TRINITY_DN16354_c1_g1_i1) was upregulated by 1.5-fold in the HPAs sample (Figure 4), and it showed high sequence similarity with other *MYB* genes involved in PA pathway, which were therefore selected as candidate genes for further analyses.

### Multiple Sequence Alignment and Phylogenetic Analysis of *OvMYBPA2* Gene

**Figure 5:**
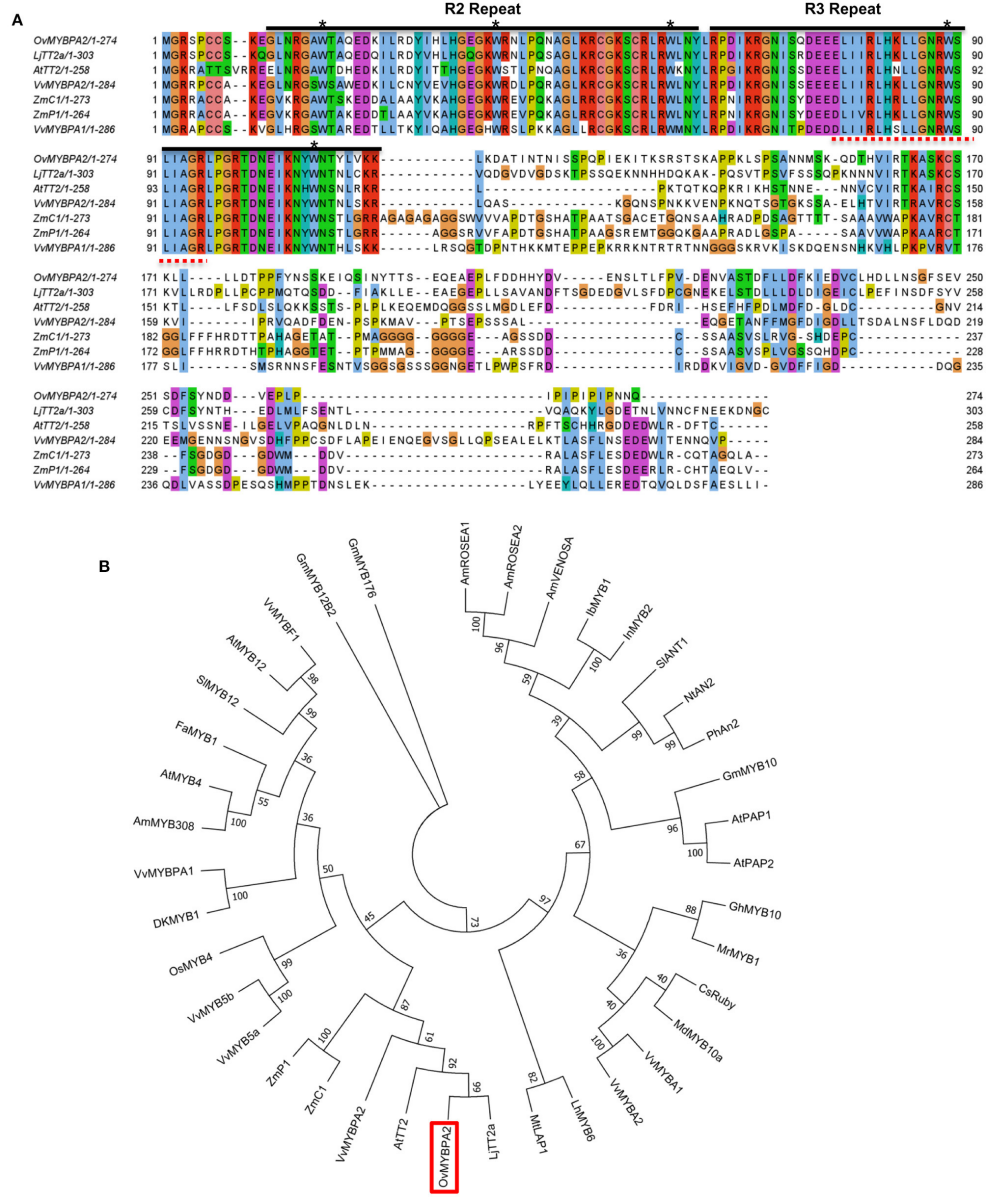
Multiple sequence alignment and evolutionary relationship of OvMYBPA2. **(A)** Alignment of amino acids sequence of *OvMYBPA2* with other MYBs (e.g., LjTT2a, AtTT2, VvMYBPA1, VvMYBPA2, ZmC1, and ZmP1) that were related to proanthocyanidins biosynthesis. The R2 and R3 repeats of MYB conserved domain were marked with black line. The five asterisks indicate conserved W residues. The bHLH-binding domains were indicated in dashed red lines **(B)** phylogenetic analysis of OvMYBPA2 and other MYB proteins related to proanthocyanidins, anthocyanidins, and flavonoid biosynthesis. OvMYBPA2 was highlighted with a red box. The sequence information for all the MYB transcription factors was downloaded from the GenBank database and listed in Supplementary Table 3.

To further characterize the function of the selected *MYB* gene, its open reading frame was cloned and named as *OvMYBPA2* (under GenBank accession number OM929200). The ORF of *OvMYBPA2* is 825 bp in length, which encodes a putative protein of 274 amino acid. The deduced OvMYBPA2 protein showed ~40% similarity to ZmC1 from maize, VvMYBPA1 and VvMYBPA2 from grape, AtTT2 from *Arabidopsis*, and 53% to LjTT2a from *L. japonicus* (Figure 5A). Multiple sequence alignment with *MYB* genes from other plant species revealed that OvMYBPA2 has a highly homologous conserved R2R3 DNA-binding domains at the N-terminus, whereas the C-terminus differed significantly in sequence and length (Figure 5A), and the bHLH-binding domain was identical to those of LjTT2a, VvMYBPA1/A2, ZmC1, and ZmP1 (Figure 5A).

OvMYBPA2 and several MYBs from other plant species that regulate PAs, anthocyanins, and flavonoids biosynthesis were used for phylogenetic analysis, and it showed that these MYBs formed three distinct clades, including the anthocyanin clade containing AtPAP1, AtPAP2, and MtLAP1, the PA clade containing AtTT2, and the flavonoid clade containing AtMYB5 and AtMYB4 (Figure 5B). OvMYBPA2 was clustered within the PA clade containing LjTT2a, AtTT2, VvMYBPA2, ZmC1, and ZmP1, suggesting that OvMYBPA2 may have a similar function in PAs regulation in sainfoin (Figure 5B).

### Over-expression of *OvMYBPA2* Gene in Alfalfa Hairy Roots

**Figure 6:**
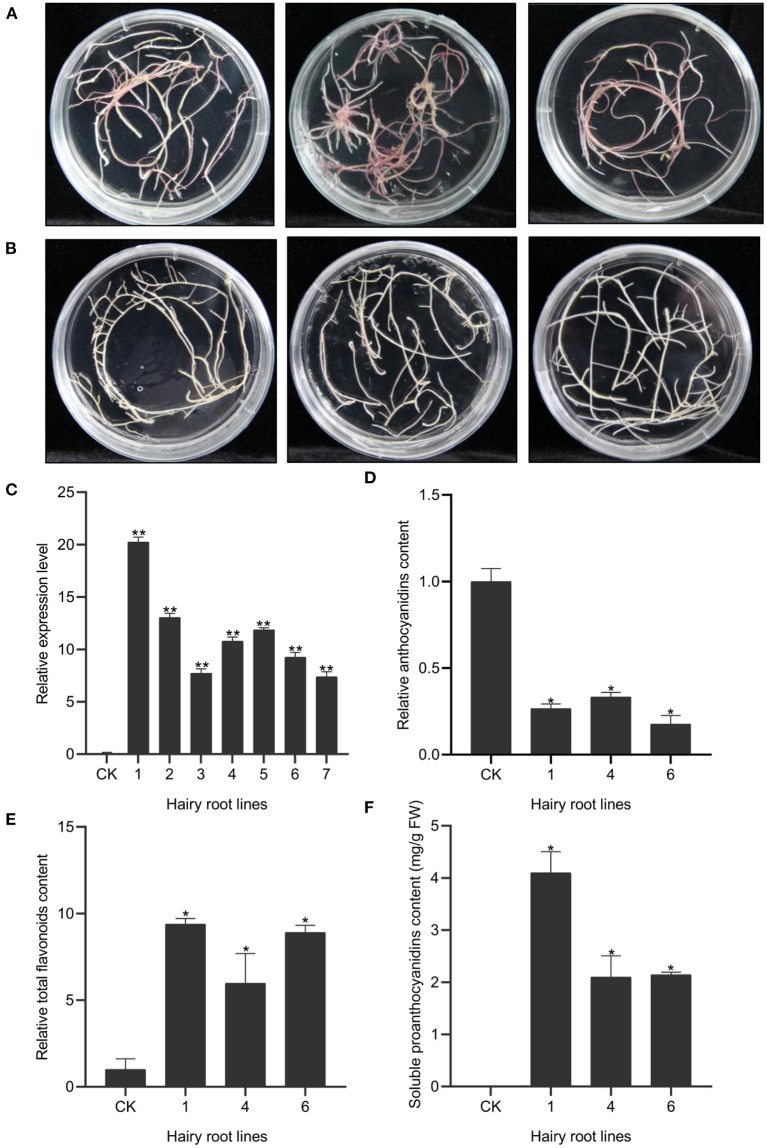
Over-expression of *OvMYBPA2* in alfalfa hairy roots. **(A)** Phenotype of control hairy roots grown on B5 medium. **(B)** Phenotype of *OvMYBPA2*-over-expressing hairy roots grown on B5 medium. **(C)** Relative expression levels of *OvMYBPA2* in transgenic hairy roots and control hairy roots (CK). **(D)** Proanthocyanidins content in different transgenic hairy root lines. **(E,F)** Relative content of anthocyanidin **(E)** and flavonoids **(F)**. Error bar depicts the standard error of mean ± SD of three biological replicates. Significance of differences are indicated with one asterisk (*p* < 0.05) and two asterisk (*p* < 0.01).

To further determine the *in vivo* regulatory function of OvMYBPA2, we over-expressed it in hairy roots of alfalfa, a target forage crop for PA bioengineering. A total of fifty-three positive transgenic hairy root lines were successfully generated and further confirmed by PCRs (Figures 6A,B). The expression level of *OvMYBPA2* was confirmed by RT-qPCR analyses in seven representative hairy root lines, but no *OvMYBPA2* expression was detected in the vector control line (Figure 6C). RT-qPCRs showed that the expression of *OvMYBPA2* was activated to various levels in different hairy roots lines as compared to the control hairy roots (Figure 6C). Among them, three lines (nos. 1, 4, and 6) with relatively high expression level and stable growth condition were propagated and selected for further analyses.

We found that anthocyanin contents in hairy root lines nos.1, 4, and 6 were significantly reduced by 73, 67, and 82% as compared to the control line (Figure 6D). Soluble PA content was 4.10, 2.10, and 2.14 mg/g FW in the hairy root line nos.1, 4, and 6, respectively, and that in the control line was negligible (Figure 6E). Total flavonoids in hairy root lines nos.1, 4, and 6 were 9.38, 5.98, and 8.90 times as compared to the control hairy root line (Figure 6F). Taken together, these data clearly indicated that over-expression of *OvMYBPA2* promoted flavonoid accumulation, in particular proanthocyanidins accumulation in alfalfa hairy roots.

## Discussion

Sainfoin is a high-quality forage grass, which is widely cultivated in western China, and it is the second legume grass after alfalfa in term of cultivation area and yield. In particular, some sainfoin contained a large amount of flavonoid, specifically PAs, which is absent in foliage of alfalfa. In this study, we analyzed flavonoid and PAs content in leaves of 46 sainfoin germplasm resources and found that soluble PA varied significantly, but total flavonoids and insoluble PAs did not show much difference (Table 1), and these data indicated that the difference in flavonoids content is mainly contributed by soluble PAs in sainfoin. This characteristic of sainfoin is distinct from the other forage crop alfalfa and its close relative *M. truncatula*, which did not accumulate PAs in the foliage tissues, but in the seed coats (Pang et al., 2007).

In addition to soluble PAs, a few flavonoid compounds were also identified, although they showed difference in composition, but their amount was not as significant as soluble proanthocyanidins (Table 1; Supplementary Figure 1). It was found that eight flavonoid compounds accumulated in both sainfoin, which was consistent with a previous report in terms of flavonoid composition (Regos and Treutter, 2010). It was notable that no proanthocyanidin monomers such as (epi)catehicn, (epi)afzenin or (epi)gallocatechins, or dimmers were detected in leaves of sainfoin by HPLC-MS in our study (Supplementary Figure 1), which was different from other plant, for examples, *Arabidopsis, M. truncatula*, or grape that accumulate free monomer of different types (Lepiniec et al., 2006; Pang et al., 2007; Chen et al., 2020b). On the one hand, these phenomena could be explained by extraction and identification method for these two representative samples. On the other hand, it is possible that a large amount of proanthocyanidin were in the form of high polymerization degree, and the identification of high molecule PAs is still challenging. Therefore, the degree of polymerization of proanthocyanidins in sainfoin requires further investigation with improved method in the near future.

With the development of molecular biology technologies, RNA-sequencing has become an effective strategy to investigate the transcriptome of non-model plant species. This method has been widely used to mine and investigate genes related to a certain pathway in plants. For example, Bai et al. analyzed the key genes related to flavonoid synthesis in *Scutellaria viscidula* using transcriptome sequencing (Bai et al., 2018), and Sun et al. mined *MYB* genes regulating flavonoid synthesis in *Zanthoxylum bungeanum* with transcriptome analysis (Sun et al., 2019). We also identified a number of genes related to isoflavonoid puerarin biosynthesis in kudzu in the previous study (Shen et al., 2021). Currently, no genome information is available for sainfoin, so this study is the first report on transcriptome sequencing of sainfoin to mine genes regulating biosynthesis of proanthocyanidins in sainfoin. A total of two sainfoins with high and low proanthocyanidin contents were used to compare the expression level of potential pathway genes, and our results showed that the transcripts of sainfoin leaves were closely related to legume plants such as *C. arietinum, M. truncatula*, and *T. pratense* (Figure 1), indicating that they may share common functional genes as they share close evolutionary relationship (Koenen et al., 2020).

The comparison of these two sainfoin resulted in the identification of more than one thousands unigenes that were differentially expressed in these two samples (Figure 2), with enriched genes in phenylpropanoid pathway and secondary metabolic pathway, and these genes are most like involved in the regulation of proanthocyanidins. Analysis of these differentially expressed genes showed that a number of *MYB, bHLH*, and *WD40* genes were differentially expressed (Figure 4; Supplementary Figures 6, 7). In particular, RT-qPCR showed that the key enzyme genes of flavonoid synthesis pathway were expressed in different tissues at different levels (Figure 3). In particular, *F3'5'H* and *ANR* genes were preferentially expressed in leaves of HPAs sainfoin, which is likely responsible for high content of PAs in leaves (Figure 3). *F3'5'H* is responsible for the hydroxylation of flavonoid in 3' and 5' hydroxy group in the B-ring, which is likely responsible for epigallocatechin and further for proanthocyanidin biosynthesis in sainfoin as previously reported (Regos and Treutter, 2010). In proanthocyanidin-rich plants like tea and grapes, *F3'5'H* gene was expanded or was expressed with relatively high level (Falginella et al., 2010; Li et al., 2014; Jin et al., 2017), which is highly correlated with proanthocyanidin accumulation. ANR is a key enzyme for the biosynthesis of epicatechins in plant, which is highly correlated with proanthocyanidins accumulation as a marker gene as reported in *M. truncatula* (Pang et al., 2007; Liu et al., 2014). Therefore, it is reasonable to deduce that differentially expressed genes could be potential candidate genes for proanthocyanidin accumulation in leaves of sainfoin.

MYB transcription factors are involved in many secondary metabolic pathways (Allan and Espley, 2018; Deng et al., 2020; Chen et al., 2021), and MYB in the MBW complex is essential for the biosynthesis of proanthocyanidins in many plant species (Li, 2014; Liu et al., 2014; An et al., 2018; Wang et al., 2019). In this study, we identified one of the differentially expressed MYB genes, *OvMYBPA2*, and found that it was expressed higher in leaves of HPAs sainfoin than in LPAs sainfoin (Figure 4), showing high sequence similarity with other MYBs involved in proanthocyanidin pathway (Figure 5), such as AtTT2 from *Arabidopsis* and LjTT2a from *L. japonicus*, these genes are the key players in the regulation of proanthocyanidins (Nesi et al., 2001; Yoshida et al., 2008), and many *MYB* genes in proanthocyanidin pathway were also applied in bioengineering of proanthocyanidins (Pang et al., 2008; Hancock et al., 2012; Gourlay et al., 2020). In the bioengineering of secondary metabolism, hairy roots are an ideal system for the characterization of many functional genes (Pang et al., 2008; Verdier et al., 2012; Hao et al., 2020). In previous studies, over-expression of *AtTT2*, or *MtPAR1*, promoted the accumulation of proanthocyanidins in hairy roots of the model legume plant *M. truncatula* (Pang et al., 2008; Verdier et al., 2012; Liu et al., 2014). Since no stable transformation is available for sainfoin, therefore, we over-expressed *OvMYBPA2* in hairy roots of alfalfa, a main target plants for proanthocyanidin bioengineering. When *OvMYBPA2* was over-expressed in hairy roots of alfalfa, anthocyanins were decreased with the increase of proanthocyanidins (Figure 6), indicating that anthocyanins were re-directed to the proanthocyanidin branch by the over-expression of *OvMYBPA2*. Besides anthocyanins, total flavonoids were also highly increased in the *OvMYBPA2* over-expression lines (Figure 6), indicating *OvMYBPA2* activated entire flavonoid to provide more flux for proanthocyanidin branch. Whether or not *OvMYBPA2* could activate proanthocyanidins in alfalfa plants besides hairy roots is currently under further investigation.

With the development of biology technology, RNA-seq has been proven to be a mature technique to mine genes related to biological functions. In this study, comparative transcriptome of two sainfoin resources revealed a number of genes related to proanthocyanidin biosynthesis in leaves, which is a golden mine for further exploration of functional genes in proanthocyanidins pathway. Besides *OvMYBPA2*, other fifty-nine *MYB* genes were also differentially expressed in two sainfoin of HPAs or LPAs, which might be the key activators or suppressors for PA biosynthesis in leaves, and the regulatory mechanism of them will be investigated further.

## Data Availability Statement

The original contributions presented in the study are publicly available. This data can be found here: NCBI, OM929200 and PRJNA810561.

## Author Contributions

ZJ and YP designed this experiment. ZJ performed the experiments and drafted the manuscript. ZJ, YL, HH, and DY analyzed experimental data. YP revised the manuscript and supervised the study. All authors have read and agreed to the published version of the manuscript.

## Funding

This project was supported by the Key Projects in Science and Technology of Inner Mongolia (2021ZD0031), the National Nature Science Foundation of China (31901386), and the Agricultural Science and Technology Innovation Program (ASTIP-IAS10).

## Conflict of Interest

The authors declare that the research was conducted in the absence of any commercial or financial relationships that could be construed as a potential conflict of interest.

## Publisher's Note

All claims expressed in this article are solely those of the authors and do not necessarily represent those of their affiliated organizations, or those of the publisher, the editors and the reviewers. Any product that may be evaluated in this article, or claim that may be made by its manufacturer, is not guaranteed or endorsed by the publisher.
