# Supplementary material for: Analyses on Flavonoids and Transcriptome Reveals Key MYB Gene for Proanthocyanidins Regulation in Onobrychis Viciifolia
Source: Front Plant Sci. 2022 Jun 24;13:941918. doi: 10.3389/fpls.2022.941918 (PMC9263696; doi:10.3389/fpls.2022.941918)
Supplement: Supplementary file 1 [file Data_Sheet_1.docx]

**Supporting Information**

**Table S1. List of accession numbers for 46 sainfoin germplasm resources used in the present study.**

| Number | Germplasm resource number | Number | Germplasm resource number |
| --- | --- | --- | --- |
| 1 | ZXY623 | 24 | ZXY06P2164 |
| 2 | ZXY777 | 25 | ZXY06P2280 |
| 3 | ZXY795 | 26 | ZXY06P2559 |
| 4 | ZXY810 | 27 | ZXY06P2642 |
| 5 | ZXY858 | 28 | ZXY06P2668 |
| 6 | ZXY883 | 29 | ZXY07P4007 |
| 7 | ZXY975 | 30 | ZXY07P4014 |
| 8 | ZXY1010 | 31 | ZXY07P4053 |
| 9 | ZXY1041 | 32 | ZXY07P4057 |
| 10 | ZXY1054 | 33 | ZXY07P4182 |
| 11 | ZXY1116 | 34 | ZXY2010P7369 |
| 12 | ZXY1154 | 35 | ZXY2010P7434 |
| 13 | ZXY1161 | 36 | ZXY2010P7456 |
| 14 | ZXY1254 | 37 | ZXY2012P9098 |
| 15 | ZXY1324 | 38 | ZXY2012P9602 |
| 16 | ZXY06P1626 | 39 | ZXY2012P9699 |
| 17 | ZXY06P1637 | 40 | ZXY2012P10295 |
| 18 | ZXY06P1725 | 41 | ZXY13523 |
| 19 | ZXY06P1751 | 42 | ZXY13687 |
| 20 | ZXY06P1830 | 43 | ZXY13709 |
| 21 | ZXY06P1838 | 44 | ZXY13865 |
| 22 | ZXY06P1994 | 45 | ZXY14172 |
| 23 | ZXY06P2132 | 46 | ZXY18657 |

**Table S2. Primer sequences used for qPCR analysis and gene cloning.**

| Name of primer | Sequences (5*'*→3*'*) | Purpose |
| --- | --- | --- |
| *OvCHI-F* | GGGACAACAACAAGCACAGC | qPCR |
| *OvCHI-R* | TCAACAAAGCCTCACCGGAA |  |
| *OvCHS-F* | TTGACATGCCTGGTGCTGAT | qPCR |
| *OvCHS-R* | TAGCACCTGCAAAACACCCT |  |
| *OvDFR-F* | TTTGATCCGGCAGTGAAGGG | qPCR |
| *OvDFR-R* | TCGGGAGATTTTGGCCTTGT |  |
| *OvF3H-F* | TTCAGCCAATTGAGGGTGCT | qPCR |
| *OvF3H-R* | CTGGTGCTGGGTTCTGGAAT |  |
| *OvF3'H-F* | TCCCAACTTGGCTCACAACT | qPCR |
| *OvF3'H-R* | GTGGGAGATTGTAGCGGTGG |  |
| *OvF3'5'H-F* | ACAACGACGCCTCCAAGAAT | qPCR |
| *OvF3'5'H-R* | CGAGGCAAGTTTAGTGGGGT |  |
| *OvANR-F* | TGCCGCGCACATATATTCCT | qPCR |
| *OvANR-R* | ACTGAGGGTATCGCTTGCTG |  |
| *OvANS-F* | TGGCGACCAAATGCAGATCA | qPCR |
| *OvANS-R* | TTGGCCGTGCCTCGTTTAT |  |
| *OvLAR-F* | TGGAGAGTTTGTCCGCGAAA | qPCR |
| *OvLAR-R* | TCTGGTCCCACACGTTGTTT |  |
| *OvMYBPA2qPCR-F* | GGCCTCAACGGACTTTCTGT | qPCR |
| *OvMYBPA2qPCR-R* | ATAGGCAACGGTTCCACGTC |  |
| *OvMYBPA2-F* | ATGGGTAGAAGCCCTTGTTGTT | cloning |
| *OvMYBPA2-R* | TTATTGGTTGTTCGGAATAGG |  |
| *actinF* | CAAAAGATGGCAGATGCTGAGGAT | qPCR |
| *actinR* | CATGACACCAGTATGACGAGGTCG |  |

**Table S3. MYB used for the phylogenetic analysis.**

| Species name | Names of MYB | Genbank Accession Numbers |
| --- | --- | --- |
| *Antirrhinum majus* | AmROSEA1 | ABB83826 |
|  | AmROSEA2 | ABB83827 |
|  | AmVENOSA | ABB83828 |
|  | AmMYB308 | P81393 |
| *Arabidopsis thaliana* | AtPAP1 | AAG42001 |
|  | AtPAP2 | AAG42002 |
|  | AtTT2 | NP_198405 |
|  | AtMYB12 | ABB03913 |
|  | AtMYB4 | NP_195574 |
| *Citrus sinensis* | CsRuby | AFB73913 |
| *Diospyros kaki* | DkMYB4 | BAI49721 |
| *Fragaria × ananassa* | FaMYB1 | AAK84064 |
| *Garcinia mangostana* | GmMYB10 | ACM62751 |
| *Gerbera hybrid* | GhMYB10 | CAD87010 |
| *Ipomoea batatas* | IbMYB1 | BAF45114 |
| *Ipomoea nil* | InMYB2 | BAE94709 |
| *Solanum lycopersicum* | SlANT1 | AAQ55181 |
|  | SlMYB12 | ACB46530 |
| *Lilium hybrid* | LhMYB6 | BAJ05399 |
| *Lotus japonicus* | LjTT2a | BAG12893 |
| *Malus × domestica* | MdMYB10a | ABB84753 |
| *Medicago truncatula* | MtLAP1 | ACN79541 |
| *Onobrychis viciifolia* | OvMYBPA2 | OM929200 |
| *Morella rubra* | MrMYB1 | ADG21957 |
| *Nicotiana tabacum* | NtAN2 | ACO52470 |
| *Oryza sativa* | OsMYB4 | BAA23340 |
| *Petunia × hybrida* | PhAn2 | AAF66727 |
| *Vitis vinifera* | VvMYBA1 | BAD18977 |
|  | VvMYBA2 | BAD18978 |
|  | VvMYBPA1 | CAJ90831 |
|  | VvMYBPA2 | ACK56131 |
|  | VvMYBF1 | ACV81697 |
|  | VvMYB5a | AAS68190 |
|  | VvMYB5b | AAX51291 |
| *Zea mays* | ZmC1 | AAA33482 |
|  | ZmPl | AAA19819 |
| *Glycine max* | GmMYB176 | NP_001236048 |
|  | GmMYB12B2 | AEC13303 |

**Table S4. List of phenol compounds identified in leaves of *O. viciifolia***

| peak | Retention  time | Mass  spectrometry | Compound | Grouping（mg/g) | |
| --- | --- | --- | --- | --- | --- |
|  |  |  |  | High  PAs | Low  PAs |
| 1 | 13.769 | 610 | quercetin 3-*O*-rutinoside | 1.175 | 1.083 |
| 3 | 14.762 | 594 | kaempferol 3-*O*-rutinoside | 0.802 | 0.407 |
| 5 | 16.262 | 302 | quercetin | 0.035 | / |
| 6 | 17.516 | 286 | kaempferol | 0.056 | 0.009 |
| 8 | 9.329 | 204 | L-tryptophan | 0.0137 | / |
| 9 | 11.262 | 338 | *p*-coumaroylquinic acid | 0.060 | 0.021 |
| 12 | 12.909 | 626 | myricetin 3-*O*-rhamnoglucoside | 0.111 | / |
| 14 | 9.889 | 354 | caffeoylquinic acid | 0.052 | / |
| 4 | 15.089 | unknown | unknown | unknown | unknown |
| 7 | 8.738 | unknown | unknown | unknown | unknown |
| 10 | 12.196 | unknown | unknown | unknown | unknown |
| 11 | 12.736 | unknown | unknown | unknown | unknown |
| 13 | 13.142 | unknown | unknown | unknown | unknown |

**Table S5. Statistic analyses of RNA-seq data from leaves of two *O. viciifolia* germplasm resources**

| Sample name | Contig numbers | Base pair (bp) | Q20% | Q30% | GC content % |
| --- | --- | --- | --- | --- | --- |
| 25-1 | 40,543,488 | 6,045,523,490 | 98.5 | 95.1 | 45 |
| 25-2 | 53,157,308 | 7,817,076,210 | 98.8 | 95.5 | 44 |
| 25-3 | 41,143,752 | 6,050,291,552 | 98.7 | 95.2 | 44 |
| 33-1 | 53,577,112 | 7,990,363,053 | 98.3 | 94.5 | 44 |
| 33-2 | 44,959,602 | 6,593,215,724 | 98.7 | 95.2 | 43 |
| 33-3 | 43,528,692 | 6,397,228,042 | 98.8 | 95.4 | 43 |

**Table S6. Statistic analyses of assembled unigenes of *O. viciifolia* leaves**

| Number | Transcripts count/length |
| --- | --- |
| Total sequence number | 52,926 |
| Total sequence base | 56,231,236 |
| Percent of GC content | 40.14% |
| Largest transcript | 14,543 bp |
| Smallest transcript | 301 bp |
| Average length | 1,063 bp |
| N50 length | 1,587 bp |

**Table S7. Statistics results of unigenes with functional annotations**

| Database | Total unigenes | Annotated unigenes | Percentage |
| --- | --- | --- | --- |
| NR | 52,926 | 37,398 | 70.6% |
| COG | 52,926 | 32,472 | 61.4% |
| KOG | 52,986 | 18,743 | 35.4% |
| KEGG | 52,926 | 17,312 | 32.7% |
| GO | 52,926 | 17,912 | 33.8% |
| Swiss-Prot | 52,926 | 28,016 | 52.9% |

**Table S8. Correlation analyses of biological triplicates of the two samples**

| Samples | 25-1 | 25-2 | 25-3 | 33-1 | 33-2 | 33-3 |
| --- | --- | --- | --- | --- | --- | --- |
| 25-1 | 1.00 | 0.96 | 0.99 | 0.98 | 0.96 | 0.94 |
| 25-2 | 0.96 | 1.00 | 0.97 | 0.95 | 0.95 | 0.93 |
| 25-3 | 0.99 | 0.97 | 1.00 | 0.98 | 0.97 | 0.96 |
| 33-1 | 0.98 | 0.95 | 0.98 | 1.00 | 0.97 | 0.94 |
| 33-2 | 0.96 | 0.95 | 0.97 | 0.97 | 1.00 | 0.97 |
| 33-3 | 0.94 | 0.93 | 0.96 | 0.94 | 0.97 | 1.00 |

**Table S9. Differentially expressed genes and their expression levels in leaves of high PAs and low PAs *O. viciifolia* samples.**

| Gene name | | Unigene number | FPKM  (High PAs) | FPKM  (Low PAs ) | Relative expression (Log_2_FC) |
| --- | --- | --- | --- | --- | --- |
| *CHS* | ***CHS-1*** | **TRINITY_DN1298_c0_g1_i1** | **11.10** | **298.09** | **-4.52** |
|  | *CHS-2* | TRINITY_DN613_c0_g1_i2 | 274.03 | 513.64 | -1.26 |
|  | *CHS-3* | TRINITY_DN20751_c0_g1_i1 | 24.32 | 51.93 | -1.65 |
|  | *CHS-4* | TRINITY_DN320_c0_g1_i4 | 138.46 | 266.88 | -1.79 |
| *CHI* | ***CHI-1*** | **TRINITY_DN24023_c0_g1_i1** | **43.46** | **1.59** | **4.62** |
|  | *CHI-2* | TRINITY_DN12115_c0_g1_i1 | 3.25 | 13.97 | -1.97 |
|  | *CHI-3* | TRINITY_DN586_c0_g2_i3 | 16.72 | 54.84 | -1.37 |
| *F3H* | ***F3H-1*** | **TRINITY_DN4402_c0_g1_i1** | **311.23** | **477.50** | **-1.11** |
|  | *F3H-2* | TRINITY_DN4145_c0_g2_i2 | 49.23 | 8.52 | 1.95 |
|  | *F3H-3* | TRINITY_DN2171_c0_g1_i1 | 522.10 | 698.33 | -0.95 |
| *F3’H* | ***F3’H-1*** | **TRINITY_DN1854_c0_g1_i3** | **14.50** | **48.97** | **-0.99** |
|  | *F3’H-2* | TRINITY_DN13740_c0_g2_i1 | 33.80 | 48.95 | -1.39 |
| *F3’5’H* | ***F3’5’H-1*** | **TRINITY_DN2008_c0_g1_i3** | **110.13** | **248.17** | **-1.82** |
|  | *F3’5’H-2* | TRINITY_DN2008_c0_g2_i2 | 54.43 | 44.52 | -0.13 |
| *DFR* | ***DFR-1*** | **TRINITY_DN282_c0_g1_i15** | **44.21** | **31.95** | **1.16** |
|  | *DFR-2* | TRINITY_DN6863_c1_g1_i9 | 6.86 | 10.20 | -0.47 |
|  | *DFR-3* | TRINITY_DN4112_c0_g2_i2 | 63.87 | 96.27 | -0.42 |
| *ANS* | ***ANS-1*** | **TRINITY_DN17670_c1_g1_i1** | **4.64** | **13.06** | **-1.26** |
|  | *ANS-2* | TRINITY_DN7309_c0_g1_i3 | 377.25 | 570.61 | -1.27 |
|  | *ANS-3* | TRINITY_DN12106_c0_g2_i1 | 10.59 | 19.20 | -0.37 |
| *LAR* | ***LAR-1*** | **TRINITY_DN511_c0_g1_i6** | **17.82** | **45.97** | **-1.52** |
|  | *LAR-2* | TRINITY_DN11234_c1_g1_i2 | 155.08 | 65.71 | 0.27 |
| *ANR* | ***ANR-1*** | **TRINITY_DN296_c0_g1_i2** | **499.79** | **661.61** | **-0.990** |
|  | *ANR-2* | TRINITY_DN231_c0_g1_i9 | 40.80 | 88.19 | -0.39 |
|  | *ANR-3* | TRINITY_DN293_c0_g2_i1 | 1.83 | 20.31 | 0.51 |

Note: Genes that were bolded were selected for further qPCR analysis in various tissues.

**A
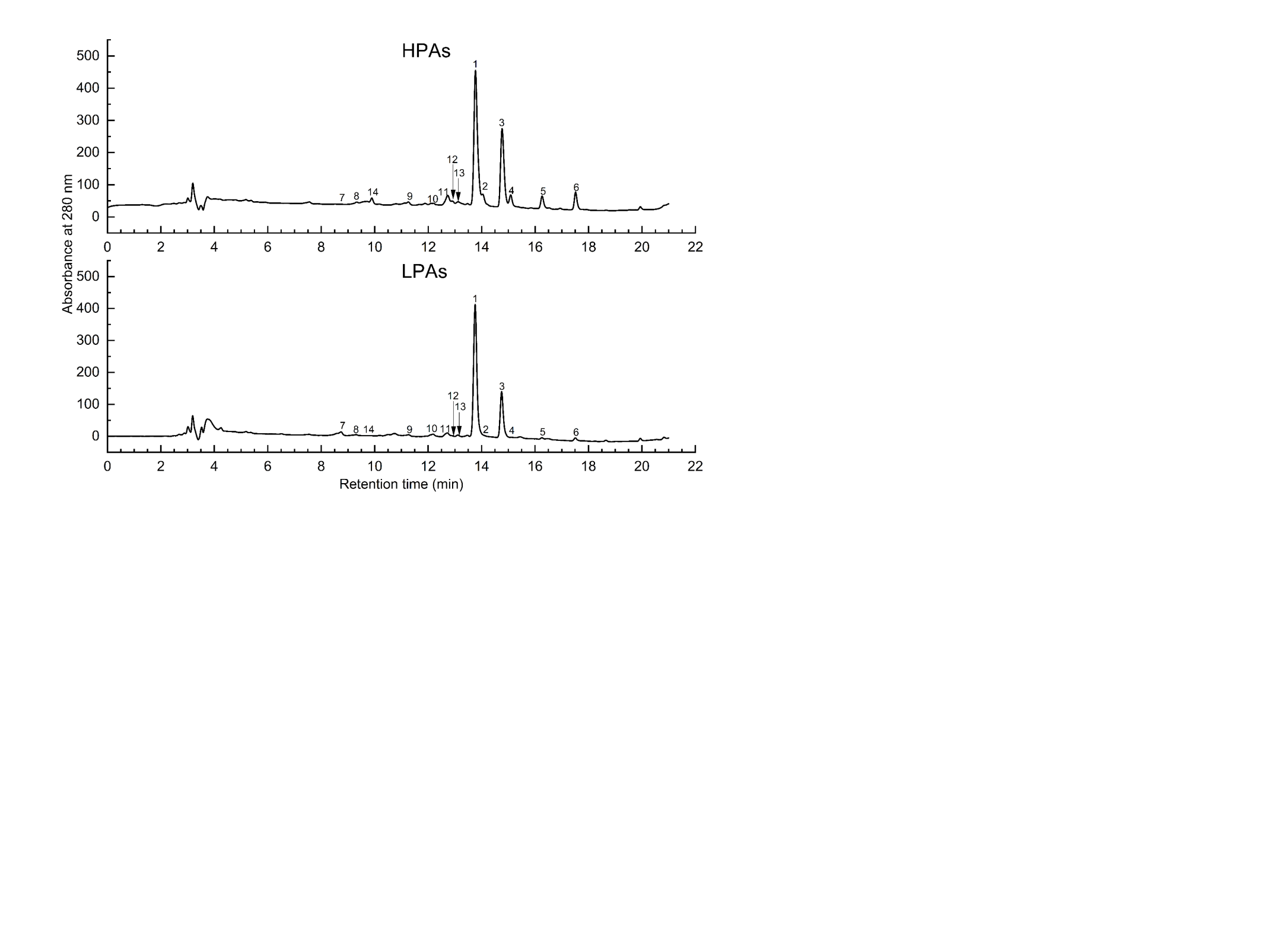
**

**B: peak1**

**C: peak 3**

**D:peak 5**

**E:peak 6**

**F:peak 8**

**G:peak 9**

**H:peak 12**

**I:peak 14**

**Figure S1.** **Identification and quantification of total flavonoids from the leaves of HPAs and LPAs samples by LC-MS.** (A) HPLC-DAD chromatograms of total flavonoids extracted from the leaves of HPAs and LPAs samples at 280 nm. (B) Mass chromatograms of eight peaks under negative iron mode. The identification of relevant components were referred to previous articles (Regos et al., 2009; Regos and Treutter, 2010), and the detailed information of these compounds were listed in Table S1.


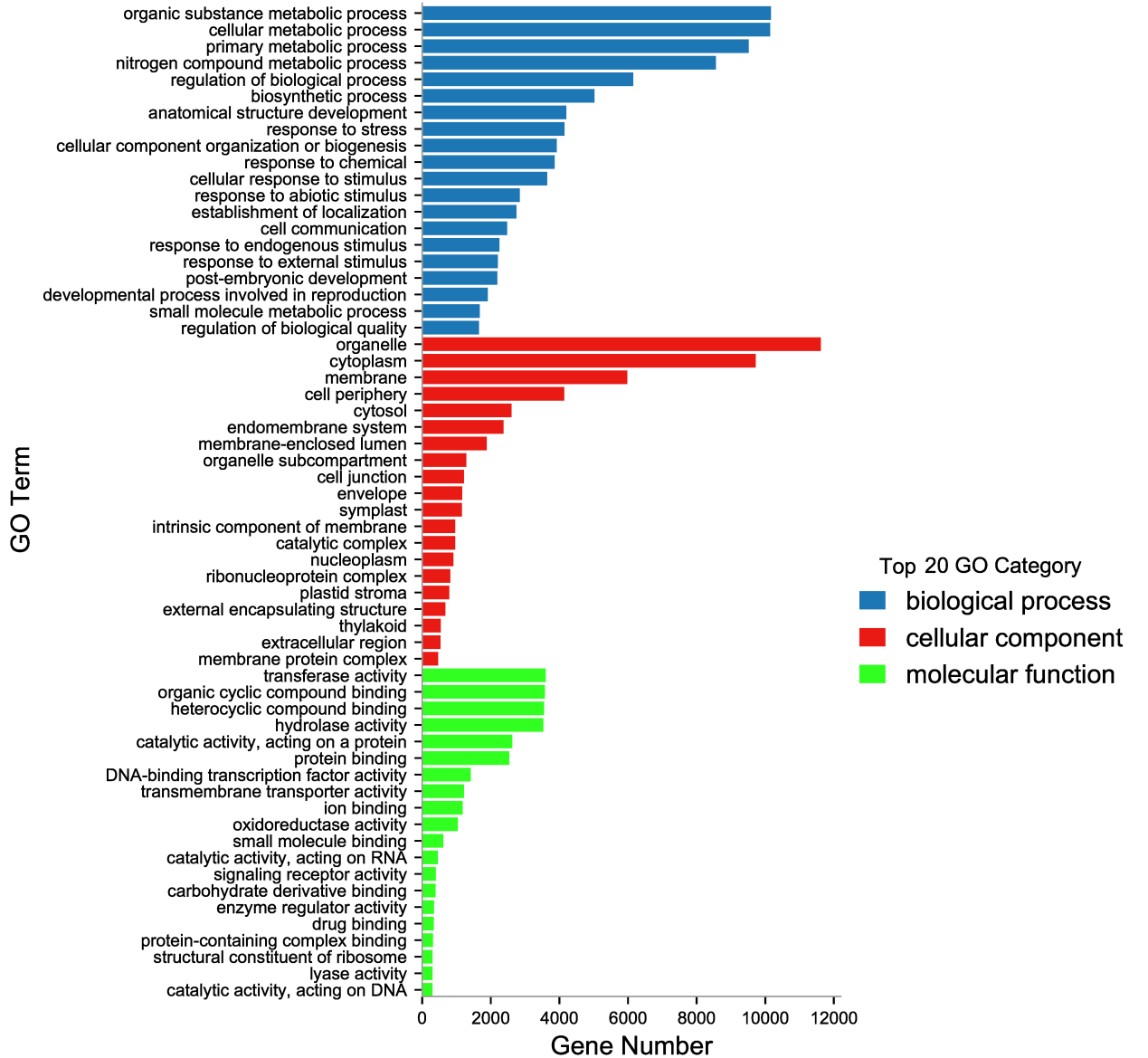


**Figure S2. GO function classification of *O. viciifolia* leave transcriptome.** All genes were classified by the following three categories: biological process, cellular component, and molecular function. The top 20 GO terms in each aspect were shown for presentation.


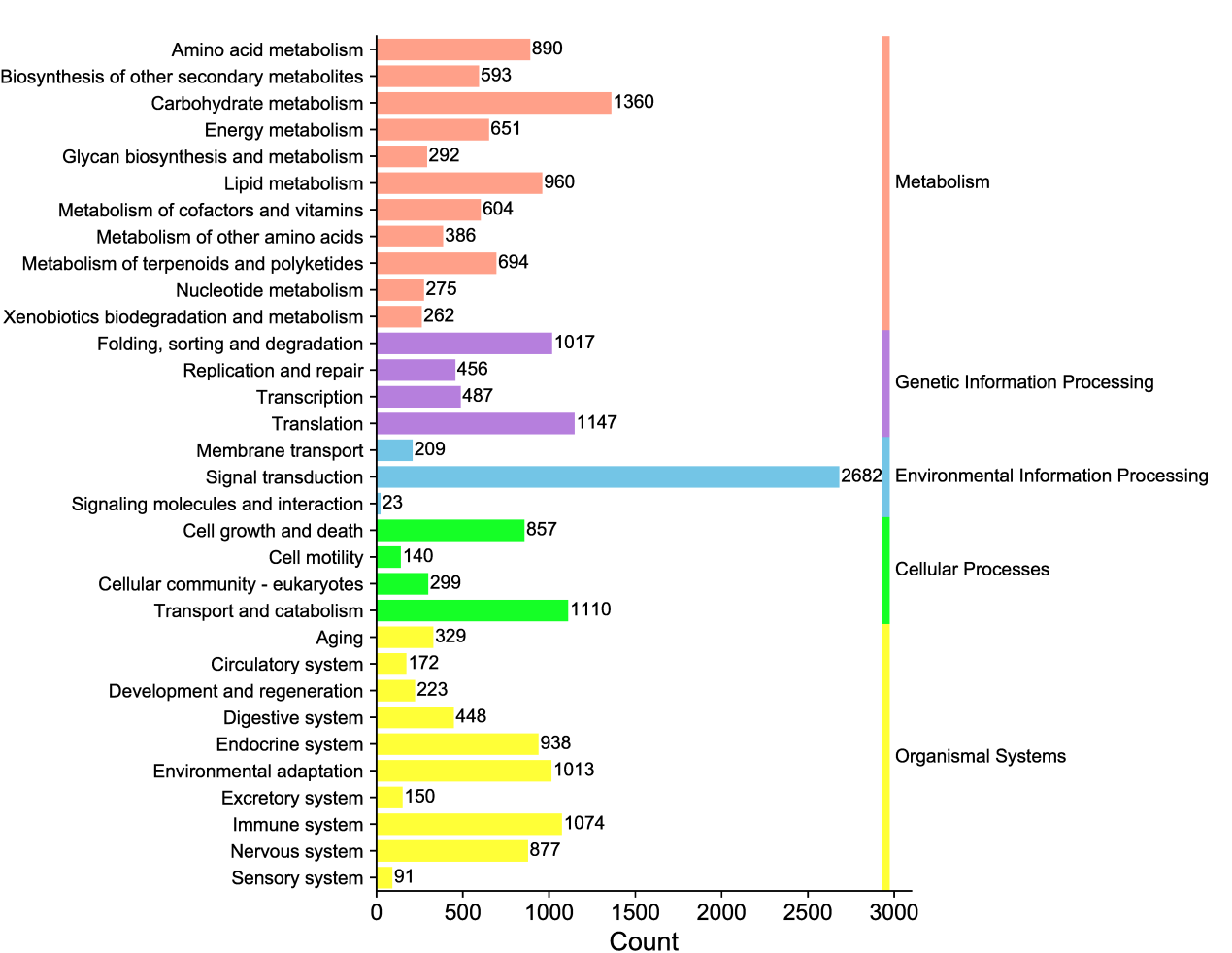


**Figure S3. KEGG pathway classification of unigenes expressed in leaves of *O. viciifolia*.** All unigenes were classified by the following five categories: metabolism, genetic information processing, environmental information processing, cellular processes, and organismal systems. Total 32 pathways were included.


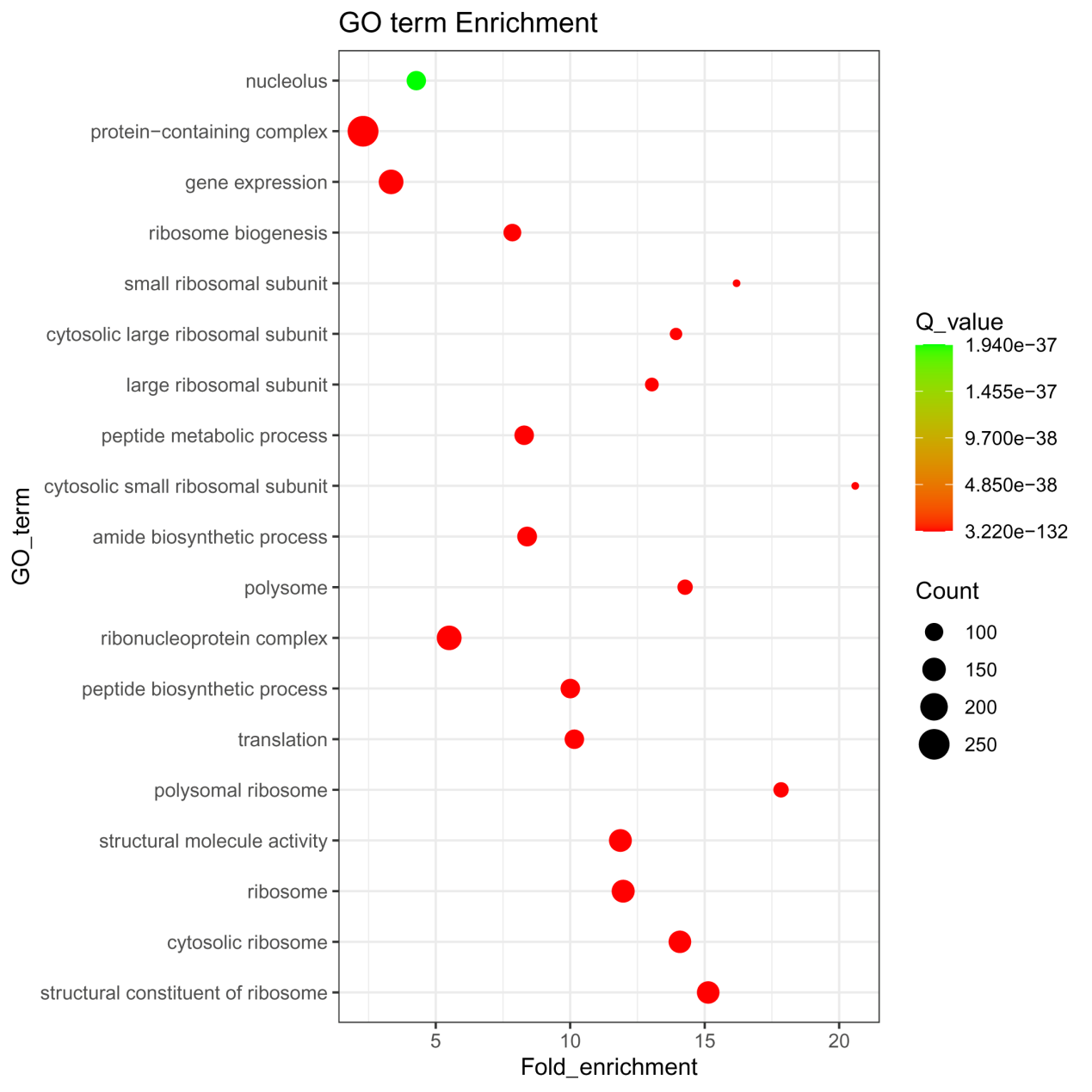


**Figure S4. Enrichment analyses of GO function of up-regulated DEGs in leaves of *O. viciifolia*.** GO function enrichment analyses were performed on 1160 up-regulated DEGs. The horizontal axis is the fold enrichment factor, and the vertical axis is the GO term. The bubbles represent the number of genes. Different colors represent significance of differences (Q-value).


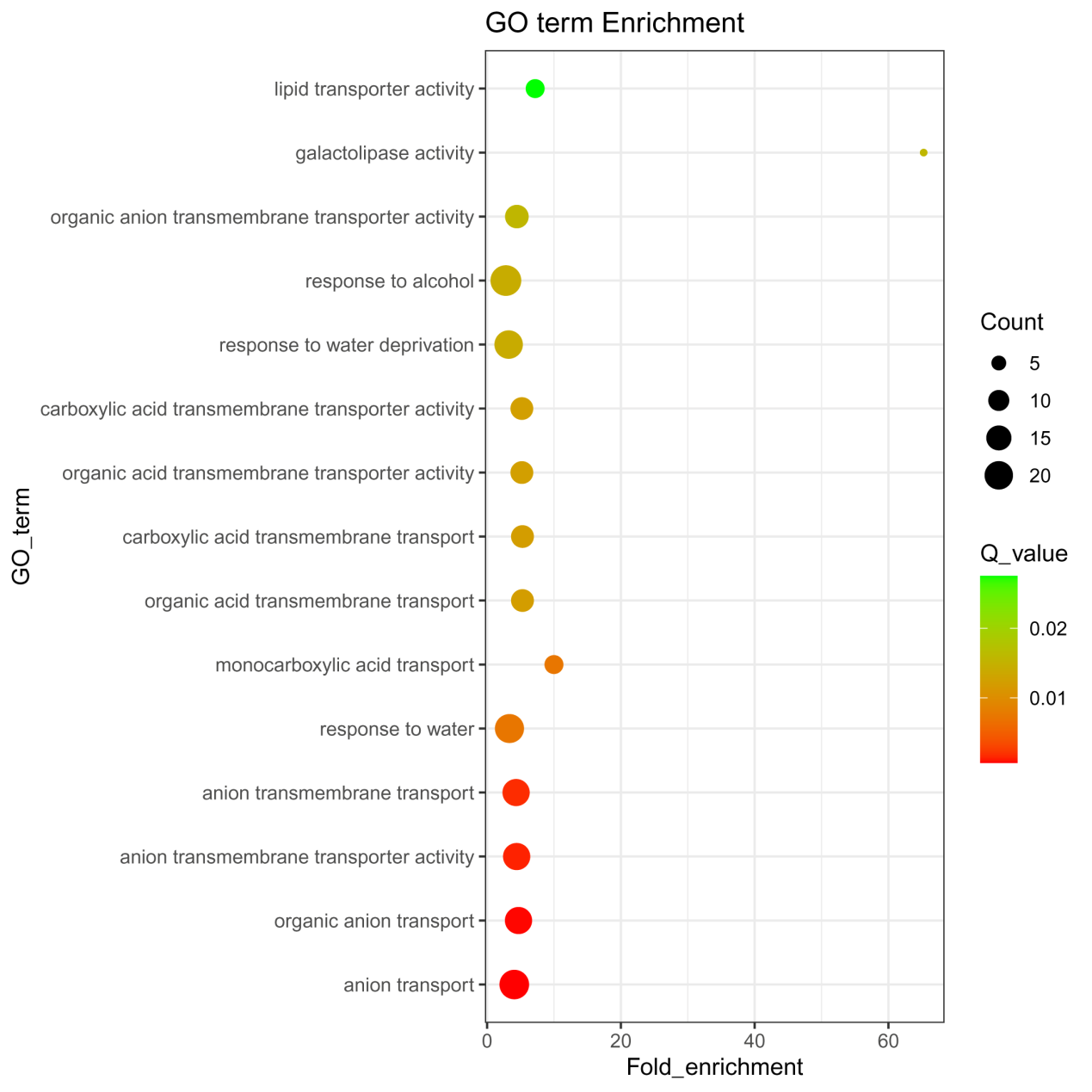


**Figure S5. Enrichment analyses of GO function of down-regulated DEGs in leaves of *O. viciifolia*.** GO function enrichment analyses were performed on 448 down-regulated DEGs. The horizontal axis is the fold enrichment factor, and the vertical axis is the GO term. The bubbles represent the number of genes. Different colors represent significance of differences (Q-value).


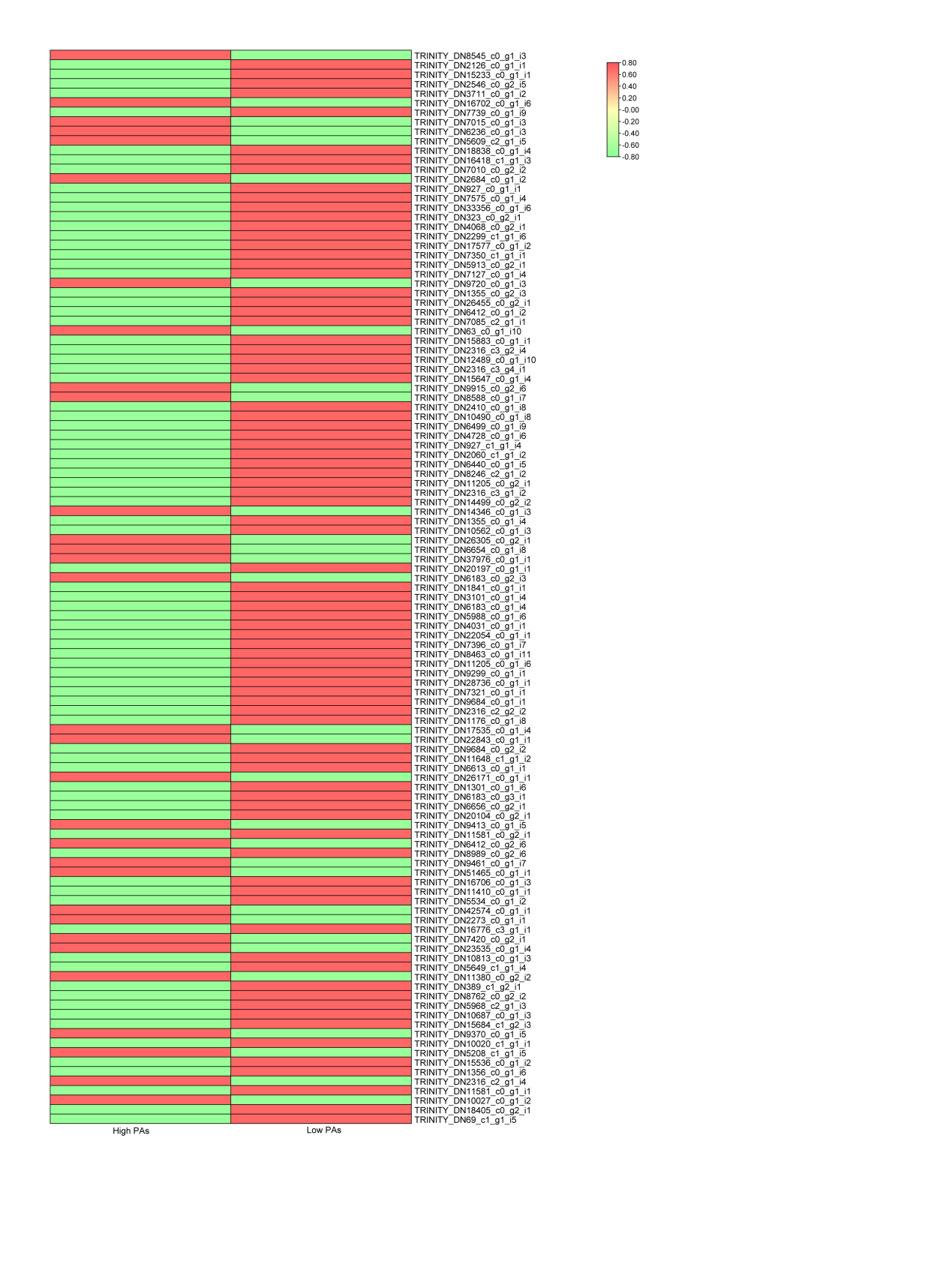


**Figure S6. Expression levels of putative *bHLH* genes in two sainfoin samples.** The heat map was drawn by TBtools with the Log_2_FPKM values of 113 bHLHs in *O. viciifolia* with high PAs and low PAs. Different colors depicts different expression levels, red means higher expression levels and green means lower expression levels.


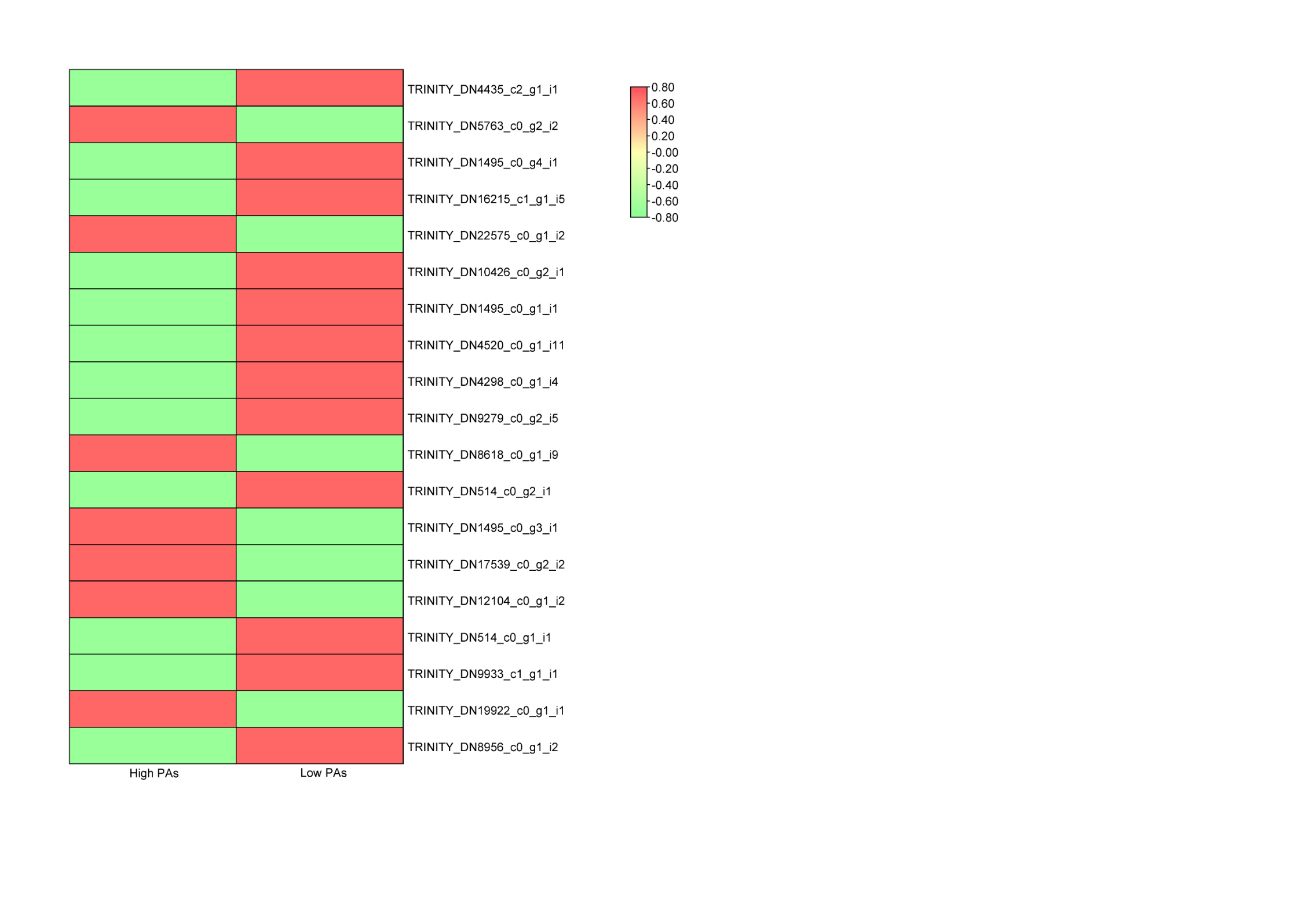


**Figure S7. Expression levels of putative *WD40* genes in two sainfoin samples.** The heat map was drawn by TBtools with the Log_2_FPKM values of 19 putative WD40 proteins in *O. viciifolia* with high PAs and low PAs. Different colors depicts different expression levels, red means higher expression levels and green means lower expression levels.
